# Dysregulation of neuroprotective astrocytes, a spectrum of microglial activation states, and altered hippocampal neurogenesis are revealed by single-cell RNA sequencing in prion disease

**DOI:** 10.1186/s40478-022-01450-4

**Published:** 2022-11-09

**Authors:** Jessy A. Slota, Babu V. Sajesh, Kathy F. Frost, Sarah J. Medina, Stephanie A. Booth

**Affiliations:** 1 One Health Division, National Microbiology Laboratory, Public Health Agency of Canada, Winnipeg, MB Canada; 2 Department of Medical Microbiology and Infectious Diseases, Faculty of Health Sciences, University of Manitoba, Winnipeg, MB Canada

**Keywords:** Prion disease, Neurodegeneration, Pathogenesis, Single-cell RNAseq, Systems biology, Microglia, Astrocytes, Neurons

## Abstract

Prion diseases are neurodegenerative disorders with long asymptomatic incubation periods, followed by a rapid progression of cognitive and functional decline culminating in death. The complexity of intercellular interactions in the brain is challenging to unravel and the basis of disease pathobiology remains poorly understood. In this study, we employed single cell RNA sequencing (scRNAseq) to produce an atlas of 147,536 single cell transcriptomes from cortex and hippocampus of mice infected with prions and showing clinical signs. We identified transcriptionally distinct populations and sub-populations of all the major brain cell-types. Disease-related transcription was highly specific to not only overarching cell-types, but also to sub-populations of glia and neurons. Most striking was an apparent decrease in relative frequency of astrocytes expressing genes that are required for brain homeostasis such as lipid synthesis, glutamate clearance, synaptic modulation and regulation of blood flow. Additionally, we described a spectrum of microglial activation states that suggest delineation of phagocytic and neuroinflammatory functions in different cell subsets. Differential responses of immature and mature neuron populations were also observed, alongside abnormal hippocampal neurogenesis. Our scRNAseq library provides a new layer of knowledge on single cell gene expression in prion disease, and is a basis for a more detailed understanding of cellular interplay that leads to neurodegeneration.

**Supplementary Information:**

The online version contains supplementary material available at 10.1186/s40478-022-01450-4.

## Introduction

Prion diseases are a rare group of fatal and infectious neurodegenerative disorders that afflict humans and animals. According to the ‘protein only hypothesis’, prion disease is caused by structural transformation of cellular prion proteins (PrP^C^) into a misfolded conformation (PrP^Sc^) that can be transmitted between individuals [67]. Prions replicate by recruiting and converting PrP^C^ into the disease-associated conformation, adding to growing amyloid fibrils that accumulate and spread throughout the brain [50, 76]. Prion accumulation is associated with brain pathogenesis that includes reactive micro- and astro-gliosis, neuronal vacuolation and synaptic dysfunction, and eventual neuronal loss, culminating in rapidly progressive neurocognitive decline [74]. While neuronal dysfunction and demise presumably cause the clinical signs and symptoms of disease, the links between prion accumulation and pathogenesis remain mysterious. A few possible explanations for neuronal susceptibility to prion infection are: (1) direct toxicity from PrP^Sc^, (2) loss of functional PrP^C^, (3) inflammation/oxidative damage from reactive astrocytes and microglia, and (4) loss of homeostatic brain cells that normally protect neurons. The complexity of prion pathogenesis makes it difficult to discern the contribution of these different possibilities towards prion neurotoxicity in vivo.

A deep understanding of molecular changes within the prion-infected brain may help identify disease-associated markers and predict the interplay between diverse brain cell subtypes that differentially respond during disease. Accordingly, transcriptional profiling of bulk brain tissues in mouse models of prion disease is a popular approach that has been widely used to describe prion pathogenesis [12, 30, 39, 48, 49, 73]. Our group has previously used microdissection of the hippocampal CA1 region, and other brain regions, combined with transcriptional profiling to associate pathological gene expression changes with precise brain regions [46, 47, 72]. Studies by Scheckel et al*.* and Kaczmarczyk et al*.* used translating ribosome affinity combined with RNAseq to profile brain-cell type specific changes in proteins actively being translated from mRNAs during prion disease [38, 70]. These studies readily identify onset of inflammatory gene expression attributed to reactive microglia and astrocytes, even at early pre-clinical stages of disease. Contrary to this, transcriptional changes associated with neuronal synaptic dysfunction are less obvious and not detected until the final clinical stages of disease.

A limitation of previous transcriptional profiling approaches to prion infection is the inability to distinguish differential responses of brain cell subtypes, hampering identification of cell type specific markers associated with disease. Here, we employed single-cell RNA sequencing (scRNAseq) of live brain cells isolated from the cortex and hippocampus of mice at the clinical stages of disease following inoculation with Rocky Mountain Laboratory strain of mouse-adapted scrapie (RML). While many neurodegenerative studies employ single-nucleus RNA sequencing [86], live single cell RNA sequencing has the advantages of wider gene coverage, unbiased transcript profiling, improved power for discriminating cell types [4], and better detection of transcriptional changes within disease-associated microglia [82]. We chose the cortex and hippocampus because they are amongst the most sensitive regions to disease-associated histological changes (neuronal vacuolation, PrP^Sc^ immunoreactivity and reactive gliosis) in this model of RML scrapie [55]. Sequencing libraries were prepared from 4 mock-infected mice collected at 110–180 days post infection (dpi) and 7 RML infected mice that reached clinical endpoint at various timepoints from 152–173 dpi. Datasets were integrated to produce a “single-cell atlas” of cortical and hippocampal brain cells, primarily consisting of microglia, astrocytes, vascular cells, oligodendrocyte progenitor cells and neurons. We identified differentially expressed transcripts within individual cell clusters, and we found many cell clusters to differ in abundance between RML and mock infected mice. In association with RML disease, we noted global decreases in relative frequencies of homeostatic astrocytes and vascular cells, and increases in oligodendrocyte progenitor cells. Additionally, we distinguished between homeostatic and disease-associated microglial (DAM) populations and identified a spectrum of microglial activation states that were associated with disease. We also examined differentially affected populations of immature and mature neurons during disease and observed evidence of abnormal neurogenesis in RML infected mice, particularly in the hippocampus.

## Materials and methods

### Mice

The Animal Care Committee of the Canadian Science Centre for Human and Animal Health approved procedures involving live animals under animal use document # H-20-024. CD1 mice were intraperitoneally inoculated with 200 µL of either RML or non-infectious 2% brain homogenate. The mice were monitored for onset of clinical signs that include dull ruffled coat, pinched abdomen and weight loss of up to 20%. Mice were sacrificed by isoflurane anesthesia followed by transcardial perfusion with PBS. Brains were immediately removed and immersed in ice-cold loading buffer (EBSS supplemented with 0.04% BSA, 0.6% glucose and 1 mM Kynurenic acid).

### Preparation of single cell suspensions

The cortex and hippocampus from both hemispheres of freshly collected brains was dissected on ice and transferred into 6-well culture dishes with dissociation solution (Hibernate e minus calcium with 20 units/mL papain and 0.005% DNase I). Tissue was crudely minced with a scalpel and dissociated for 40 min at 37 °C by swirling the culture dishes every 3–4 min. A single cell suspension was made by gently triturating the tissue with a serum-coated p1000 pipette tip 10 times. At this point, cell suspensions were kept on ice and all centrifugation steps were performed at 4 °C. Large debris was allowed to settle for 2 min before transferring supernatant to a new 15 mL falcon tube. Debris was further removed using Debris removal solution (Miltenyi Biotech) according to manufacturer’s instructions. Briefly, the cell pellet was re-suspended in 1550 µL of loading buffer, mixed with 450 µL of debris removal solution, overlayed with 2 mL of loading buffer, centrifuged at 3000×*g* for 10 min and the top 2 layers were removed. The remaining cells were washed once in 5 mL of loading buffer before removing myelin using Myelin Removal Beads II (Miltenyi Biotech). Briefly, the cell pellet was re-suspended in 270 µL of loading buffer, mixed with 30 µL Myelin Removal Beads II, incubated for 15 min on ice, washed in 2700 µL of loading buffer and bead-labelled cell suspensions were passed through the magnetic field of a MACS separator using LS columns (Miltenyi). The flow through (containing myelin-freed cells) was retained, passed through a 70 µm MACS strainer (Miltenyi) and cells were re-suspended in a final volume of 50–100 µL of loading buffer before counting via trypan blue staining with a hemocytometer.

### 10× genomics single cell RNA sequencing

Single cell sequencing libraries were prepared from 10,000 cells using the Chromium Next GEM Single Cell 3ʹ Reagent Kits v3.1 (Dual Index) (10× Genomics) according to manufacturer’s instructions. Briefly, single cell suspension made from the cortex and hippocampus were diluted into cDNA reaction mixtures to sequence 10,000 cells using the provided table from 10× genomics. The reaction mixture and gel beads were loaded onto Chromium Next GEM Chip G (10× genomics) before partitioning on a Chromium Controller. The resulting GEMs were further processed into sequencing libraries according to the manufacturer’s protocol without modification, except 15 cycles were used for the final PCR amplification step. Libraries were dual indexed using Dual Index Plate TT Set A and unique indices were used for each library. Quality of the amplified cDNA and final sequencing libraries was assessed using Bioanalyzer High Sensitivity DNA kits (Agilent) on a Bioanalzyer2000 instrument prior to sequencing. Libraries were sequenced to a minimum depth of 30,000 reads per cell on an Illumina NextSeq 2000 instrument using P3 reagents (100 cycles) with the recommended read configuration from 10× genomics (Read 1–28 cycles; i7 Index—10 cycles; i5 Index—10 cycles; Read 2–90 cycles).

### Data analysis

Illumina sequencing reads were first pre-processed using the cellranger pipeline from 10× genomics. Raw bcl files were de-multiplexed using cellranger mkfastq, and cellranger count was then used to align fastq files to the mouse mm10 reference genome and count known transcripts.

Each dataset was then independently quality controlled by removing ambient RNA reads, filtering low quality cells and removing doublets. The R package SoupX [87] was used to remove ambient RNA reads directly from the cellranger output using the default method of automatically estimating the contaminating fraction of UMIs. The following filtering criteria was then applied to remove low quality cells: n genes > 1000, % mitochondrial genome reads < 20, % ribosomal protein reads > 1, percent hemoglobin reads < 20. Finally, doublets were removed using the R package DoubletFinder [52] with default settings (pN = 0.25 and pK = 0.09), including the estimation of 7.6% doublets for 10,000 cells as indicated by 10 × genomics.

The QC’d datasets were then normalized and integrated using the Seurat (v4) [27, 78] R package. Datasets were first normalized independently using the SCTransform function of Seurat, employing the "glmGamPoi" Gamma-Poisson Generalized Linear Model and regressing out the percentage of mitochondrial reads. Integration anchors between datasets were then identified with Seurat using reciprocal PCA analysis. One Mock (Mock48CX) and one RML (RML145CX) dataset were used as the reference for rPCA. Seurat was then used to integrate all datasets clustering cells using the default graph-based clustering approach and default UMAP dimensionality reduction approach. Cell clusters were then classified by brain cell type using the SCType [31] R package with a customized database of brain cell marker genes. The top marker genes of each cell cluster was also determined using the FindAllMarkers() function of Seurat and were inspected manually to make a final cell-type determination for each cluster. Cell clusters were classified as either astrocytes, microglia, perivascular macrophages, oligodendrocyte progenitor cells, glutamatergic neurons, immature neurons, endothelial cells, pericytes, vascular smooth muscle cells, vascular leptomeningeal cells, lymphocytes, or ependymal cells.

### Statistics

Marker genes were identified using Seurat using the default method of identifying differentially expressed genes between two groups of cells using a Wilcoxon Rank Sum test. *p* values were adjusted with the bonferroni correction using all genes in the dataset. Differentially expressed genes were identified by the following criteria: FDR *p* value < 0.05, |log2FC|> 0.5 and pct.1-pct.2 > − 0.1 or < 0.1 for increased/decreased genes respectively. This was used to identify differentially expressed genes between cell clusters, and between RML and Mock cells within individual clusters. Non-parametric Mann–Whitney U tests were used to compare the proportions of each cell cluster in the cortex and hippocampus between RML and Mock infected mice. We had limited statistical power when comparing cell populations between RML and Mock infected mice (only two hippocampal cell populations from Mock infected mice), so we applied a relaxed cutoff of *p* value < 0.1 to distinguish cell-population changes that were associated with disease. Hierarchical clustered heatmaps were prepared using the ComplexHeatmap[23] R package using the default Pearson distance method. Gene ontologies enrichment analysis of provided gene lists was performed with Enrichr [14, 43]. Enrichr was also used to identify lists of specific transcripts that were driving enrichment of gene ontologies and were mentioned throughout the text.

## Results

### A single cell atlas of prion disease in the murine cortex and hippocampus

**Fig. 1:**
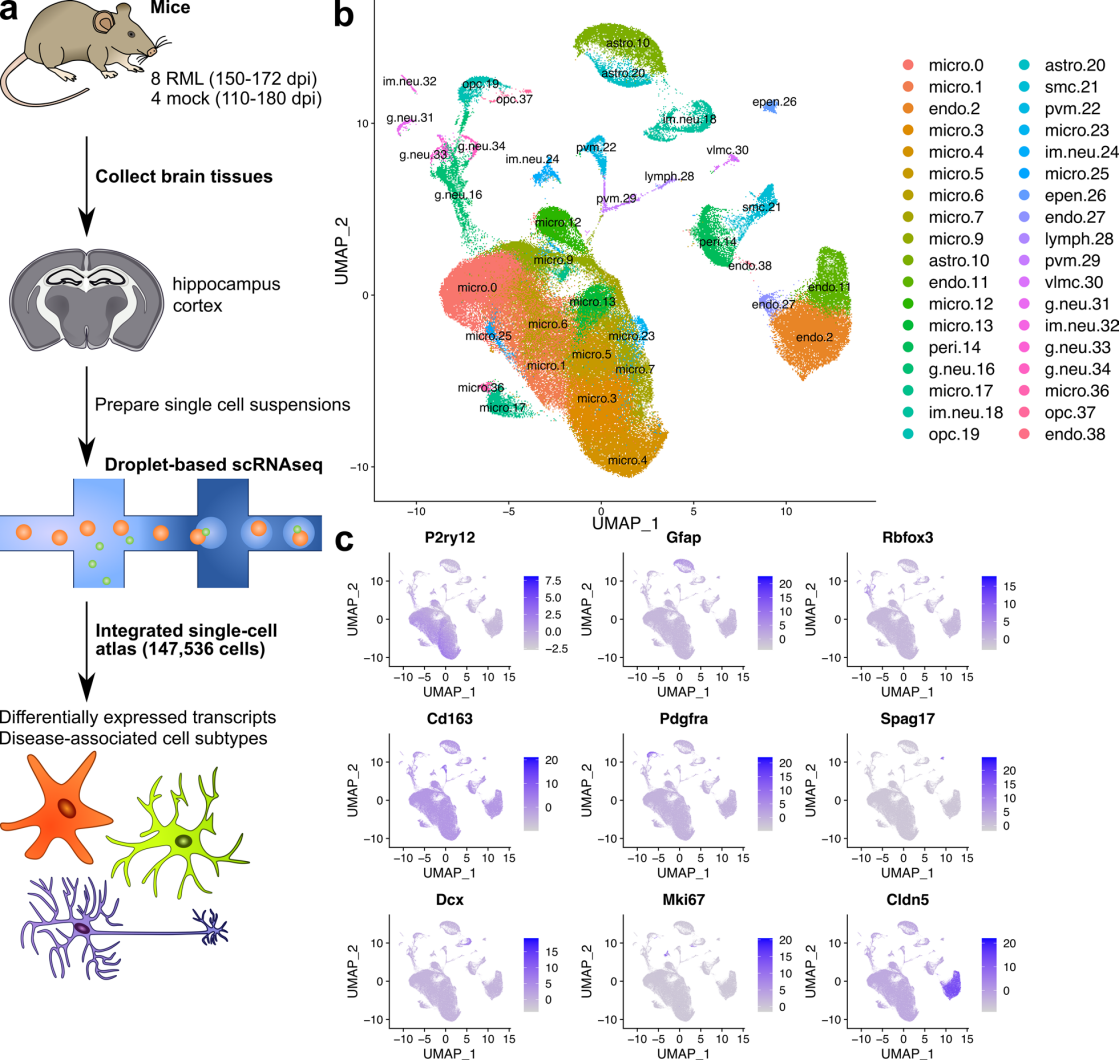
A single cell atlas of brain cells from the cortex and hippocampus from mice infected with RML scrapie or mock infection. **A** Schematic representation of workflow for single-cell sequencing of cortical and hippocampal cells during prion disease. **B** UMAP projection of all 147,536 cells sequenced from the cortex and hippocampus of RML and mock infected mice. Cells were clustered using graph based clustering and cell types were assigned to each cluster using SCType (Additional file 4) followed by manual inspection of marker genes (Additional file 2) identified for each cluster. The total number of cells isolated from RML and mock infected mice that were assigned to each cluster is provided in Additional file 3. **C** Normalized expression level of canonical marker genes for major brain cell types were plotted on the UMAP projection to verify identities of each cell type. These include *P2ry12* (microglia), *Gfap* (astrocytes), *Rbfox3* (mature neurons), *Cd163* (perivascular macrophages), *Pdgfra* (oligodendrocyte progenitor cells), *Spag17* (ependymal cells), *Dcx* (immature neurons), *Mki67* (neural progenitor cells) and *Cldn5* (vascular cells). micro—microglia; endo—vascular endothelial cells; astro—astrocytes; peri—pericytes; g.neu—mature glutamergic neurons; im.neu—immature neurons; opc—oligodendrocyte progenitor cells; smc—vascular smooth muscle cells; pvm—perivascular macrophages; epen—ependymal cells; lymph—lymphocytes; vlmc—vascular leptomeningeal cells

To profile the response of brain cell sub-populations to prion disease, we performed single cell RNA sequencing of cortical and hippocampal cells isolated from 8 RML infected mice when they reached clinical endpoint criteria at time points ranging from 150–172 dpi (Fig. 1A). For comparison, we also sequenced cortical cells from 4 mock mice collected at 147, 168, 186 and 189 dpi and hippocampal cells from 5 mock mice at 110, 147, 168, 186 and 189 dpi. We provide information on the mouse number, brain region, treatment and number of days post infection for each sequencing library included in our analysis in Additional file 1: Table S1. We were unable to exactly match the ages of Mock and RML mice used because we processed cortical and hippocampal brain tissues from one mouse per day. This was done to ensure consistency in the preparation of single-cell suspensions from all mice used in the study. Reagent clogs in the microfluidics of the chromium controller during separation of single cells reduced the usable dataset in the case of the hippocampal samples to 7 RML and 2 mock mice. These two mock mice used were collected at 110 and 147 dpi. Following pre-processing and quality control, the resulting 21 single-cell RNAseq datasets were integrated to produce an “atlas” of brain cells during prion infection, consisting of 147,536 cells that were classified into 39 transcriptionally distinct clusters via Seurat’s graph based clustering approach (Fig. 1B).

We used automated classification of brain cell types with SCType (based on custom reference markers; Additional file 4) combined with manual inspection of marker genes (Additional file 2) to assign a cell type identity for each cluster. We noted that 3 of the clusters (8, 15 and 35) had unusually high expression of genes associated with technical-artefacts (e.g. high mitochondrial gene expression, *Malat1* etc.) [15, 32]. Consequently, we could not identify clear brain cell type specific markers, so we removed these clusters from the final atlas. In total, we identified populations of astrocytes, microglia, perivascular macrophages, oligodendrocyte progenitor cells, glutamatergic neurons, immature neurons, endothelial cells, pericytes, vascular smooth muscle cells, vascular leptomeningeal cells, lymphocytes, and ependymal cells. We verified the identities of these cell types by examining the expression of the canonical marker genes *P2ry12* (microglia), *Gfap* (astrocytes), *Rbfox3* (mature neurons) *Cd163* (perivascular macrophages), *Pdgfra* (oligodendrocyte progenitors), *Spag17* (ependymal cells), *Dcx* (immature neurons), *Mki67* (neural progenitors) and *Cldn5* (endothelial cells) (Fig. 1C). Mature oligodendrocytes were absent from the dataset as expected, given the use of myelin removal beads to minimize clogs in the microfluidics of the chromium controller during cell separation. We then used these clusters representative of cellular sub-types to characterize differences related to brain pathobiology during prion infection. Given its relevance to disease, we examined the expression of *Prnp* across the single cell atlas, and found it to be most highly expressed by astrocytes, neurons, and surprisingly, ependymal cells (Additional file 1: Fig. S1).

### Transcriptional changes and altered cell sub-type composition during prion disease

**Fig. 2:**
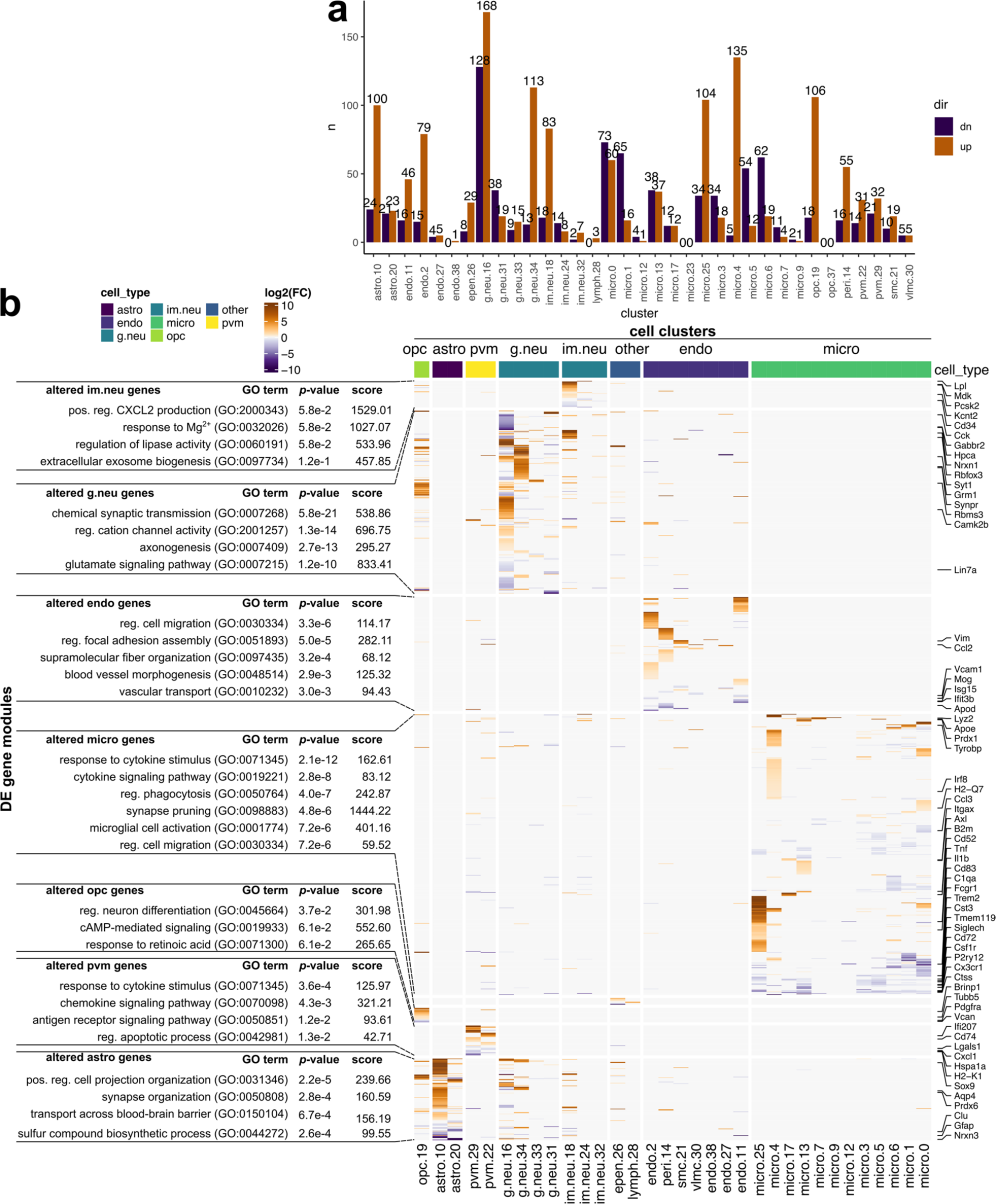
Differentially expressed transcripts within each individual cluster of cells isolated from RML and mock infected mice. **A** Number of transcripts that met differential expression criteria within each cluster when comparing cells isolated from RML and mock infected mice. Differentially expressed transcripts were defined by: FDR *p* values  < 0.05, |Log2 fold change|> 0.5, > 25% cell expression and 10% increased/decreased cell expression for increased/decreased transcripts respectively. **B** Hierarchical clustering of Log2 fold changes for all differentially expressed transcripts within each cluster. The full list of differentially expressed transcripts is provided as Additional file 8. astro—astrocytes; endo—endothelial cells; g.neu—mature glutamergic neurons; im.neu—immature neurons; micro—microglia; opc—oligodendrocyte progenitor cells; pvm—perivascular macrophages; epen—ependymal cells; lymph—lymphocytes; pos. —positive; reg. —regulation of

To characterize transcriptional responses to prion disease, we performed differential expression analysis between all cells isolated from prion-infected versus mock-infected mice within each cluster independently (Fig. 2). According to criteria of FDR *p* values  < 0.05, average |log2 fold change|> 0.5 and %cell-expression differences > − 0.1 or < 0.1 for increased/decreased genes respectively, we identified differentially expressed transcripts within most of the cell-type-specific clusters (Fig. 2A, Additional file 8). Further examination via hierarchical clustering of log2 fold change values within each cluster revealed the majority of transcriptional changes in response to prion disease showed little overlap between the different clusters, and we concluded that most were specific to cell-types or sub-types. (Fig. 2B).

**Fig. 3:**
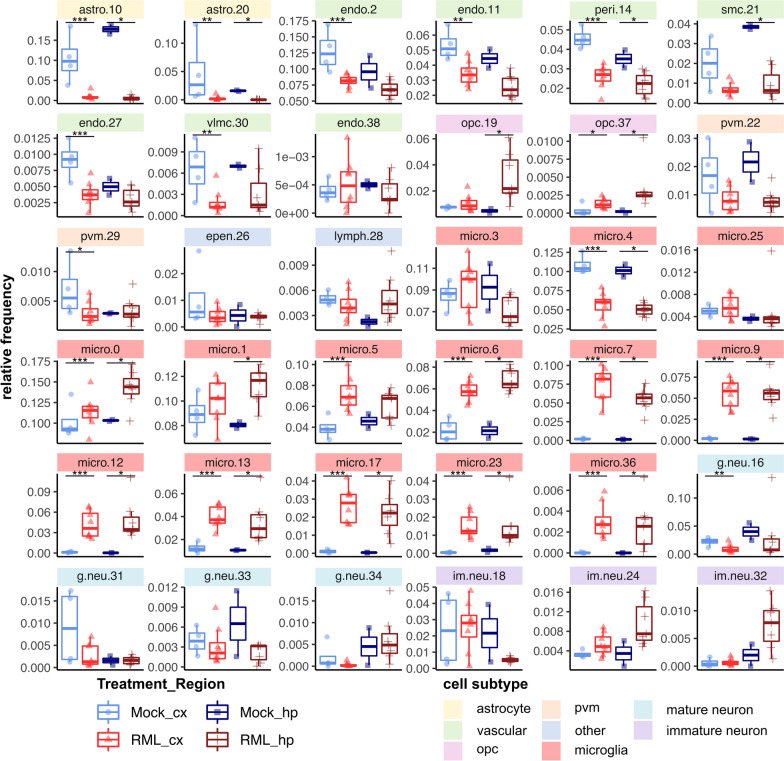
Differences in relative frequency of cell populations isolated from the cortex and hippocampus of RML infected mice compared to mock infected mice. The relative frequency of each cell cluster is plotted for the cortex and hippocampus of RML and mock infected mice. *p* values s were calculated using the non-parametric Mann–Whitney U tests. * *p* values  < 0.1, ** *p* values  < 0.05, *** *p* values  < 0.01. All *p* values are provided in Additional file 5. astro—astrocytes; endo—endothelial cells; g.neu—mature glutamergic neurons; im.neu—immature neurons; micro—microglia; opc—oligodendrocyte progenitor cells; pvm—perivascular macrophages; epen—ependymal cells; lymph—lymphocytes; cx—cortex; hp—hippocampus

In single cell RNAseq, cells are assigned to specific clusters entirely based on their transcriptomes. Therefore, transcriptional responses to prion disease might also be reflected by differences in the relative frequency of cell clusters between prion- and mock-infected mice. In other words, differences in the relative frequency of sub-clusters of a given cell-type might indicate transitions from one transcriptional state to another during disease. Therefore, we examined the relative proportion of cells assigned to each cluster within the cortex and hippocampus from either RML infected- or mock-infected mice (Fig. 3, Additional file 5). Altogether, there were some striking differences in the relative composition of various cell-types associated with RML disease. Differences in relative frequency can also be influenced by changes to absolute cell count that occur during disease. In the context of prion disease, this is expected for reactive microglia and astrocytes that are well known to proliferate and for vulnerable neurons that decline in number due to cell death [74]. Technical factors can also influence the observed relative frequency, such as difficulties in cell dissociation, cell death during preparation of single-cell suspensions, and liberation of individual cells from debris or cell-to-cell contacts. Therefore, it is challenging to interpret whether the relative frequencies we present for each cell cluster reflect transitions of transcriptional status, or absolute challenges in cell count. Nonetheless, we present these relative frequencies because in many cases, this metric provided clues into the response of brain cell-types to prion disease. Additionally, the low sample size of *n* = 2 and *n* = 7 respectively for the Mock-hippocampus and RML-hippocampus groups was a limitation for distinguishing statistically significant disease-associated differences in relative frequency of hippocampal cell transcriptomes. Despite this, many changes in relative frequency were common between the hippocampus and cortex and were more reliable.

### Vascular dysfunction implicated through abnormal transcription during prion disease

The blood brain barrier is comprised of various vascular cells including endothelial cells, pericytes, smooth muscle cells and vascular leptomeningeal cells [17]—all of which were present in our single cell atlas (Fig. 1). Disruptions to the blood brain barrier are a common feature of aging and neurodegeneration [42]. Consistent with this, prion-altered vascular transcripts were enriched in ontologies related to cell migration, blood vessel morphogenesis and vascular transport (Fig. 2B, Additional file 8). In addition to these transcriptional changes within vascular cell clusters, we also noticed that most vascular cell clusters decreased in relative frequency in association with disease (Fig. 3). We did not observe evidence of cell-death related transcription by vascular cells during prion disease, and so we cannot conclude whether the decrease of vascular cells were related to blood brain barrier breakdown. It is possible that we observed decreased vascular cell frequencies because of microglial proliferation or other technical factors. Many of the prion altered vascular were overexpressed by clusters endo.2, endo.11 and peri.14. Specific examples of notable disease-altered transcripts that represent enriched gene ontologies are listed as follows: Cluster endo.2 overexpressed transcripts related to cell migration (*Sema5a*, *Flt1*, *Lef1*, *Pecam1*, *Gcnt2*, *Rhoc*, *Dock1*, *Ptk2*, *Rab11a*, *Igf1r*) and angiogenesis (*Sema5a*, *Ramp2*, *Flt1*, *Rock1*, *Rhoj*, *Tek*, *Ism1*). Cluster endo.11 overexpressed transcripts related to blood brain barrier transport (*Slco1c1*, *Slc16a1*, *Slc2a1*, *Mfsd2a*). Cluster peri.14 overexpressed transcripts related to actin filament/supramolecular fiber organization (*Carmil1*, *Myo1b*, *Ubb*, *Col8a1*, *Myh11*, *Mfge8*, *Aldoa*, *Svil*, *Eps8*). These signatures of abnormal transcription seem to hint of vascular dysfunction or remodeling that might occur during prion disease, with blood brain barrier transport possibly being altered. Inflammation is well known to cause disruptions to brain vascular cells [42], so we were not surprised to observe evidence of vascular dysfunction in our single cell atlas. However, our analysis cannot determine whether blood brain barrier permeability is altered at the phenotypic level during prion disease, and so more detailed studies are required to investigate this hypothesis.

### Oligodendrocyte progenitor cells are transcriptionally modulated during prion disease

Transcriptional alterations to oligodendrocytes are rarely the focus of investigations into prion pathogenesis because mature oligodendrocytes are considered relatively resistant to prion replication [66]. However, mature Olig2^+^ oligodendrocytes were recently shown to decrease at the advanced stages of disease in a murine model of Creutzfeldt-Jakob disease [3], implying a role in pathogenesis. In our dataset, populations of oligodendrocyte progenitors were increased in relative frequency during RML disease, particularly in the hippocampus (Fig. 3). This is consistent with a previous bulk RNAseq analysis, where we inferred increased oligodendrocyte progenitors through increased *Pdgfra* abundance during RML disease [72]. However, it is also possible that we observed the increase in oligodendrocyte progenitor frequency due to technical reasons such as resistance to cell death during isolation relative to other cell types in the condition of prion disease. Disease-altered oligodendrocyte progenitor transcripts were enriched in ontologies related to neuron differentiation, cAMP signaling and response to retinoic acid and included downregulation of canonical oligodendrocyte progenitor cell markers *Pdgfra* and *Vcan* (Fig. 2B, Additional file 8). These transcripts were disease-altered in cluster opc.19, but not opc.37. A few disease-altered transcripts were also shared between oligodendrocyte progenitor cells with neurons and astrocytes. Notable upregulated disease-altered oligodendrocyte progenitor transcripts were related to cell adhesion (*Kirrel3*, *Ptprt*, *Tenm1*, *Unc5d*, *Lrrc4c*, *Dscaml1*, *Cdh8*, *Cdh18*), synapse assembly (*Gabrb3*, *Kirrel3*, *Dnm3*, *Gabrb2*, *Farp1*, *Ppfibp1*, *Lrrc4c*, *Ppfia2*) and neurotransmission (Gria2, *Neto1*, *Dlgap2*, *Gria3*, *Grin3a*). Notable downregulated oligodendrocyte progenitor transcripts were related to gap junctions (*Ptprd*, *Cntnap2*, *Agt*) and nervous system development (*Cntnap2*, *Vcan*, *Cntn4*, *Adgrl3*). These findings suggest that transcriptional dysfunction oligodendrocyte progenitor cells is an underappreciated component of prion pathogenesis—an avenue that is worthy of further investigation.

### Transcriptional changes of microglia and perivascular macrophages during prion disease

Microglia made up the largest population of cells (99,756/147,536 = 67%) assigned in our library and are well described as particularly responsive to prion replication, taking on reactive phenotypes that can both exacerbate pathology through excess inflammatory signaling and can protect against disease through clearance of toxic PrP^Sc^ [53, 58, 63]. As expected, altered microglial transcripts were involved in cytokine signaling, phagocytosis and microglial activation (Fig. 2B, Additional file 8). Surprisingly few markers of reactive microglia were increased within individual microglia clusters, although there were a few such as *Lyz2*, *Apoe*, *Tyrobp* and *Irf8*. Some microglia-specific markers were decreased across some of the individual microglial clusters, such as *P2ry12*, *Tmem119*, *Csf1r*, and *Cx3cr1*. Homeostatic markers like *P2ry12* and *Tmem119* are generally reported to decrease in reactive microglia [41, 54]. Similar to microglia, prion altered transcripts within perivascular macrophages were related to inflammatory signaling through cytokines, chemokines and antigen receptors. There was little overlap between prion-altered transcripts of microglia and perivascular macrophages, implying a distinct response to prion disease. Unsurprisingly, some of the most drastic changes in cellular populations during RML disease were seen in microglia (Fig. 3). We noticed a few of the microglial clusters either decreased, or did not change in relative frequency, whereas many of the microglial clusters increased in abundance and we suspected that these corresponded to reactive, or “disease-associated” microglia that are well known to increase during disease [74]. Unlike microglia, we did not observe an expansion of perivascular macrophages in disease, and perivascular macrophages (pvm.22 and pvm.29) appeared to decrease in the hippocampus and cortex. This could possibly reflect clustering of activated perivascular macrophages with the disease associated microglia, or cellular migration to other brain regions.

### Distinct transcriptomes reveal a spectrum of microglial activation states during prion disease

**Fig. 4:**
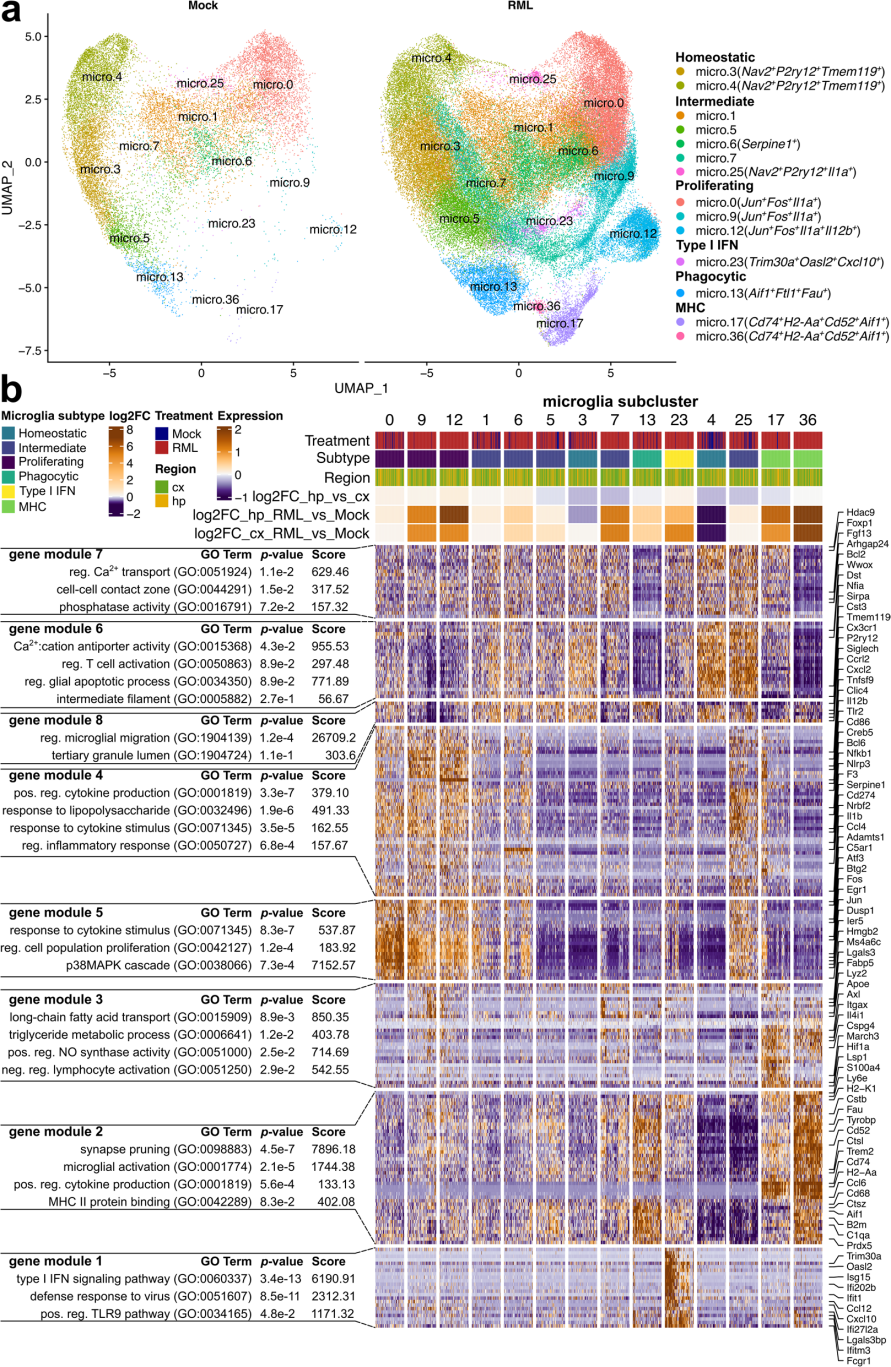
Microglia take on a spectrum of activation states in association with RML disease. **A** UMAP projections of all 99,756 microglial cells isolated from RML and mock infected mice. **B** Hierarchical clustering of all marker genes identified within each microglial sub-cluster. Marker genes were grouped using K-means clustering, and each gene cluster was functionally annotated with enriched GO terms using Enrichr. micro—microglia; cx—cortex; hp—hippocampus

Nearly 100,000 microglial transcriptomes were sequenced, making our single cell dataset particularly well suited to characterize the diversity of microglial activation states during prion disease. Furthermore, compared to single nucleus sequencing, our live single-cell sequencing approach can improve detection of transcriptional changes within activated microglia [82]. Therefore, to define transcriptional states of individual microglia subtypes, we subset the dataset to include only the 99,756 microglial transcriptomes (Fig. 4A). We retained the original microglial clusters from the full atlas, and did not perform further sub-clustering. When comparing microglia isolated from infected with mock- infected mice, we could see that some microglial clusters were nearly unique to disease (micro.9, micro.12, micro.17, micro.23, and micro.36) and were strongly associated with disease (Fig. 4A).

**Fig. 5:**
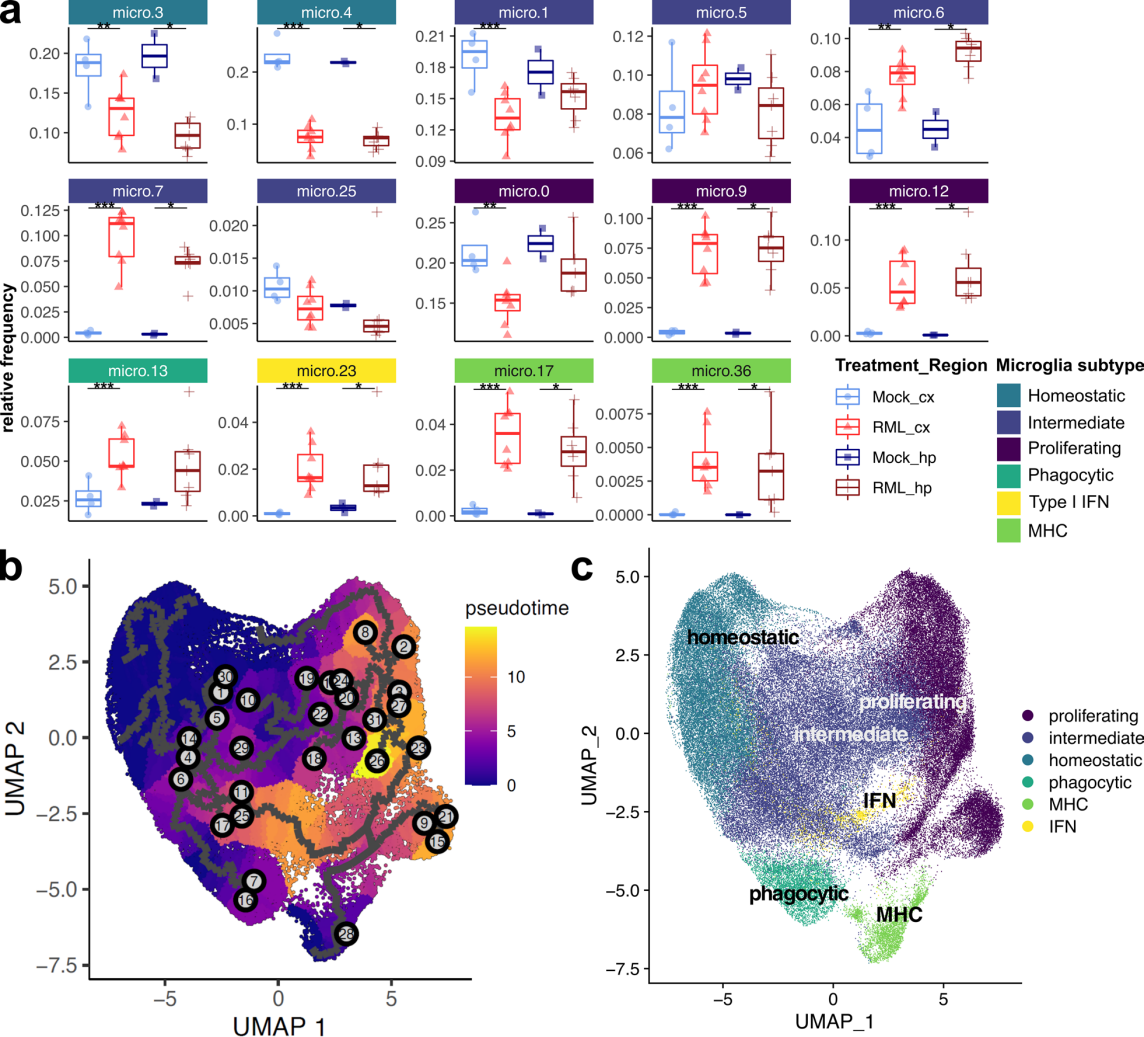
Monocle trajectory analysis of microglia categorized into 5 transcriptional subtypes. **A** The relative frequency of each microglia sub-cluster is plotted for the cortex and hippocampus of RML and Mock infected mice. *p* values s were calculated using non-parametric Mann–Whitney U tests. * *p* values  < 0.1, ** *p* values  < 0.05, *** *p* values  < 0.01. All *p* values are provided in Additional file 9. **B** UMAP projection plot of all 99,756 microglial transcriptomes with color mapping to transcriptional pseudotime calculated with Monocle. Cells from cluster micro.4 were supplied to serve as the root for calculating transcriptional pseudotime and trajectories. **C** UMAP projection plot with color mapping to microglial subtypes

We next functionally characterized the microglial subtypes by identifying marker genes highly expressed by each cluster, up to a maximum of 25 per cluster, resulting in 218 transcripts supplied for hierarchical clustering (Fig. 4B). K-means clustering of genes was used to classify the marker transcripts into 8 gene modules that were functionally annotated with representative enriched gene ontologies. Altogether, we found that reactive microglia take on a spectrum of transcriptional states characterized by expression of genes important for various aspects of glial functionality such as phagocytosis, or cytokine signaling. Based on expression of these gene modules (Fig. 4B), and the expression of specific microglial marker transcripts (Additional file 1: Fig. S2), we further classified microglia into 5 subtypes: (1) homeostatic, and the following reactive subtypes: (2) proliferating, (3) phagocytic, (4) type I interferon (IFN) responding, and (5) antigen presenting (MHC). These functional subtypes are similar to what has previously been reported in relation to Alzheimer’s disease [16]. We also classified some of the microglial clusters as representing intermediate transcriptional states between these subtypes. Within the subset microglial dataset, the homeostatic microglial clusters decreased in relative frequency, whereas reactive microglial clusters (proliferating, phagocytic, IFN and MHC subtypes) increased in relative frequency in association with disease (Fig. 5A, Additional file 9). This could indicate conversion of homeostatic microglia into reactive forms. Therefore, we performed a monocle trajectory analysis of the microglial cells to measure transcriptional status as a function of gene “pseudotime” by supplying cells from cluster micro.4 to serve as the “root” for the trajectory (Fig. 5B and C). We noted a circular transcriptional trajectory between homeostatic microglia, intermediately activated microglial states, and proliferating microglia with branches into phagocytic, antigen-presenting and interferon-responding microglial populations. Altogether, our interpretation was that microglia form a continuous spectrum of transcriptional states, where multiple possible transcriptional trajectories can allow homeostatic microglia to reach distinct disease-associated reactive states, thus mirroring the complexity of functionally distinct phenotypes observed in the brain.

### Transcriptional signatures of five microglial subtypes associated with prion disease

Homeostatic microglia (micro.3 and micro.4) were marked by high expression of the canonical microglial markers *P2ry12*, *Cx3cr1, Tmem119* in addition to *Nav2*, a marker of microglia under healthy conditions [75] (Additional file 1: Fig. S2). These microglia had high abundance of gene modules 6, 7 and 8 that were enriched in ontologies related to calcium transport (*Cacnb2, Bcl2, Ank2*), regulation of glial apoptosis (*Prkca*) and regulation of microglial migration (*P2ry12*, *Cx3cr1*) (Fig. 4A). Homeostatic microglia elicited a disease-associated decrease in relative frequency (Fig. 5A), although this does not necessarily indicate that the absolute cell count of homeostatic microglia decreases in the prion infected brain. Given the possible transcriptional trajectories that could allow homeostatic microglia to reach different reactive states (Fig. 5B and C), this decrease in relative frequency likely corresponds to conversion of homeostatic microglia into intermediate and eventually reactive transcriptional states during disease.

Gene modules 4 and 5 were involved in cytokine signaling, regulation of inflammation and regulation of cell proliferation and were highly expressed by proliferating microglia (micro.0, micro.9 and micro.12) (Fig. 4A) that were demarked by high expression of *Jun*, *Fos* and *Il1a* (Additional file 1: Fig. S2). Together, *Jun* and *Fos* encode proteins that form the AP1 transcription factor that induces inflammatory gene expression in microglia [84]. Interestingly, cluster micro.12 was uniquely marked by very high expression of the cytokine *Il12b* (Additional file 1: Fig. S2). Furthermore, *Cd14* was highly expressed by micro.9 and micro.12 and is a co-receptor for LPS that modulates inflammatory signaling, important for microglial responses to tissue damage-associated signals [35]. We also noted an expansion of proliferating microglial clusters micro.9 and micro.12 in association with disease (Fig. 5A). Altogether, these results suggest that proliferating *Jun*^+^*Fos*^+^ microglia might contribute towards inflammatory cytokine signaling during prion infection. Specific examples of cytokine signaling related transcripts expressed by these microglia include *Cd86, Egr1, Pdgfb, Fos, Ptgs2, Cxcl2, F3, Nfkb1, Socs3, Bcl6, Il1b, Ccl4, Il12b, Tnfsf9,* and *Junb*.

Phagocytic microglia (micro.13) were marked by high expression of *Aif1*, *Ftl1* and *Fau* (Additional file 1: Fig. S2) and highly expressed gene module 2 that was enriched in synapse pruning and microglial activation, containing many microglial activation markers (Fig. 4B). Specific examples of classical microglial activation markers expressed by these microglia include *C1qa, C1qb, C1qc, Tyrobp, Trem2, Aif1, B2m, Prdx5, Fcer1g, Cstb, Ctsz, Cd63,* and *Cd68*. Given that this corresponds to a classic signature of reactive microglia, we were not surprised to see that the relative frequency of micro.13 increased in association with disease (Fig. 5A). Interestingly, *Aif1*^+^*Ftl1*^+^ microglia corresponded morphologically dystrophic iron-accumulating microglia in an Alzheimer’s mouse model [40], possibly providing clues as to the role of phagocytic microglia in prion disease.

Antigen presenting microglia (micro.17 and micro.36) highly expressed phagocytosis related genes (gene module 2, also highly expressed by the phagocytic microglia cluster micro.13) and genes important for antigen presentation (Fig. 4B). These microglia were marked by high expression of *Cd74*, *H2-Aa*, *Cd52* and *Ccl6* (Additional file 1: Fig. S2). The antigen presentation genes that were highly expressed by these microglia were *Cd74, H2-Aa, H2-Eb1, H2-Ab1, H2-K1,* and *H2-D1*. Clusters micro.17 and micro.36 showed some of the most dramatic increases in relative frequency in association with disease (Fig. 5A) and were nearly absent the Mock mice. In fact, micro.36 was not detected at all in the hippocampal cells isolated from Mock mice and was only detected in one cortical cell suspension of Mock mice. Therefore, we postulate that these antigen-presenting microglia subtypes represent highly activated reactive microglia that are strongly associated with prion disease. *Cd74* is thought of as a marker of M1 microglial activation, and is expressed by highly activated microglia in the diseased-brain [37, 79], supporting this notion.

*Trim30a*, *Oasl2* and *Cxcl10* were highly expressed by type I interferon responsive microglia (micro.23, Additional file 1: Fig. S2) that highly expressed gene module 1 (Fig. 4). Examples of type I interferon responsive transcripts expressed by these microglia include *Ifitm3, Bst2, Rsad2, Isg15, Ifit1, Gbp2, Ifit3, Ifit2,* and *Cxcl10*. Like the other reactive microglia subtypes, the type I interferon responsive microglia also show a strong disease-associated increase in relative frequency (Fig. 5A). Type I interferon signaling is often thought of as detrimental in the context of brain pathology, but a recent study has suggested that this pathway might protect neurons during prion infection [33].

As expected, microglia that were considered to represent intermediate transcriptional states (micro.1, micro.5, micro.6, micro.7, and micro.25) had varying expression of the different microglial gene modules (Fig. 4B). Interestingly, micro.6 was uniquely marked by very high expression of *Serpine1* (Additional file 1: Fig. S2)—an inhibitor of tissue plasminogen activator (tPA) that promotes microglial migration and inhibits phagocytosis in vitro [36]. Intermediate microglial clusters micro.6 and micro.7 were both positively associated with disease through increases in relative frequency (Fig. 5A).

### Dysregulation of neuroprotective astrocytes during prion disease

Astrocytes are one of the main cells types responsible for brain homeostasis through neurotransmitter uptake/recycling, potassium buffering, metabolism, and protection against oxidative stress among other neuroprotective functions [6]. In the context of prion disease however, astrocytes are one of the first cells to take on active phenotypes during disease that may have various beneficial or detrimental roles, concomitant with the earliest detectable deposits of PrP^Sc^ [81]. We observed a striking global decrease in relative frequencies of astrocyte populations associated with RML disease (Fig. 3) and to our surprise; we did not observe a clearly resolved cell cluster corresponding to reactive astrocytes. Disease-altered astrocyte transcripts were enriched in ontologies related to synapse organization, blood brain barrier transport and sulfur biosynthesis, hinting at modulation of astrocyte homeostasis functions (Fig. 2B, Additional file 8). Most of the disease-altered astrocyte transcripts were upregulated in cluster astro.10, and notable transcripts were the reactive astrocyte marker *Gfap*, and transcripts related to cell junction assembly (*Kirrel3*, *Gpm6a*, *Farp1*, *Cdh2*, *Cdh20*, *Ctnnd2*, *Nrcam*, *Cdh19*), sulfur metabolism (*Bcan*, *Gstm1*, *Angpt1*, *Cspg5*, *Prelp*, *Chsy3*, *Gstm5*) and cell projection organization (*Ntrk2*, *Fut9*, *Magi2*, *Il1rapl1*, *Atp1b2*, *Prkd1*). We also noticed a notable group of transcripts downregulated in cluster astro.20 that were related to axonogenesis (*Robo2*, *Auts2*, *Nrxn3*, *Slit2*). This signature of differential transcription indicates dysfunction of the homeostatic astrocytes that were captured by our live single cell approach.

**Fig. 6:**
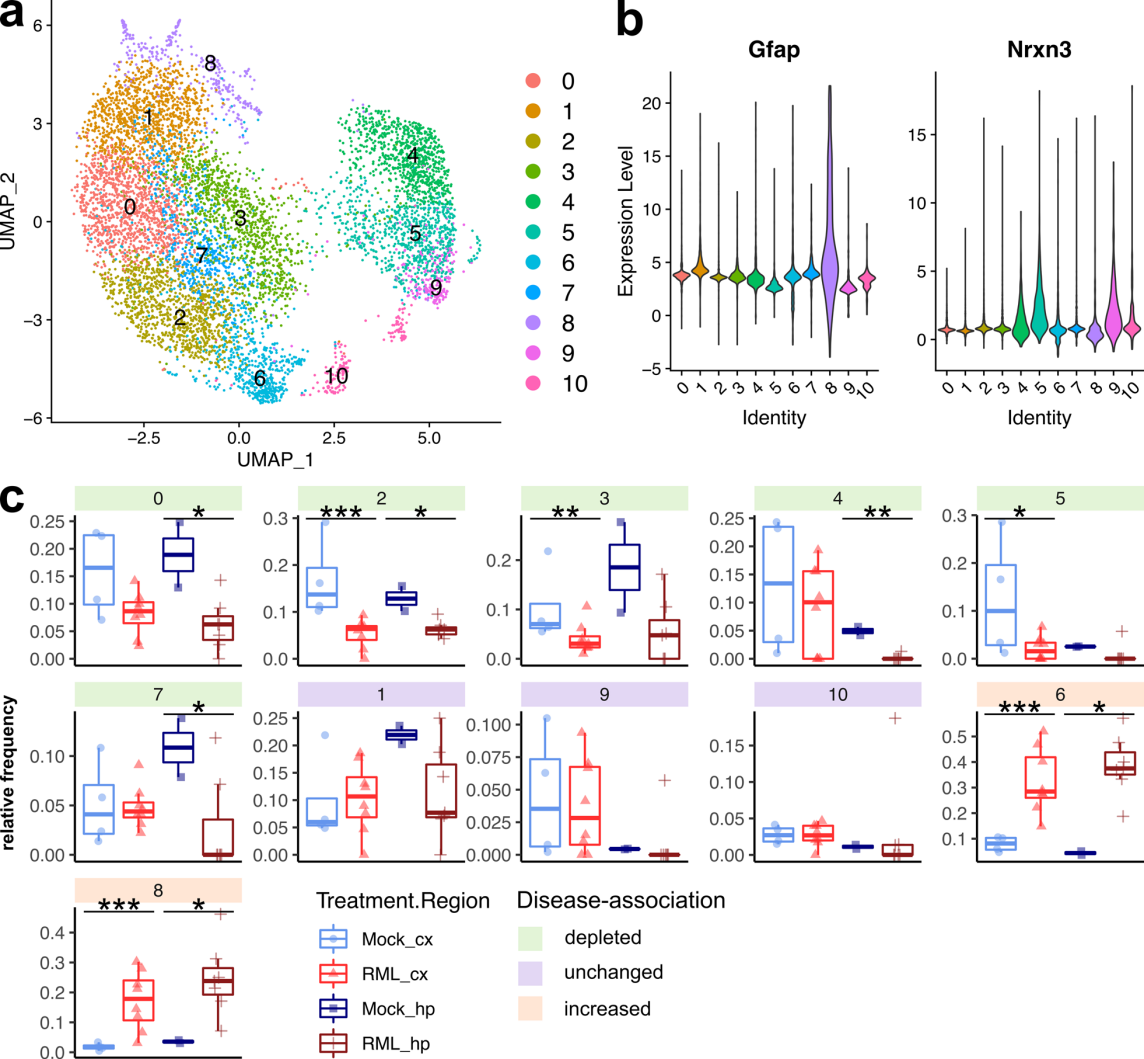
Sub-cluster analysis of astrocytes reveals disease-depleted and disease-associated subtypes during prion disease. **A** UMAP projection of all 7,813 astrocytes categorized into 11 sub-clusters. **B** Violin plots showing expression of *Gfap* and *Nrxn3* across each astrocyte sub-cluster. **C** The relative frequency of each astrocyte sub-cluster is plotted for the cortex and hippocampus of RML and Mock infected mice. *p* values s were calculated using non-parametric Mann–Whitney U tests. * *p* values  < 0.1, ** *p* values  < 0.05, *** *p* values  < 0.01. All *p* values are provided in Additional file 6

**Fig. 7:**
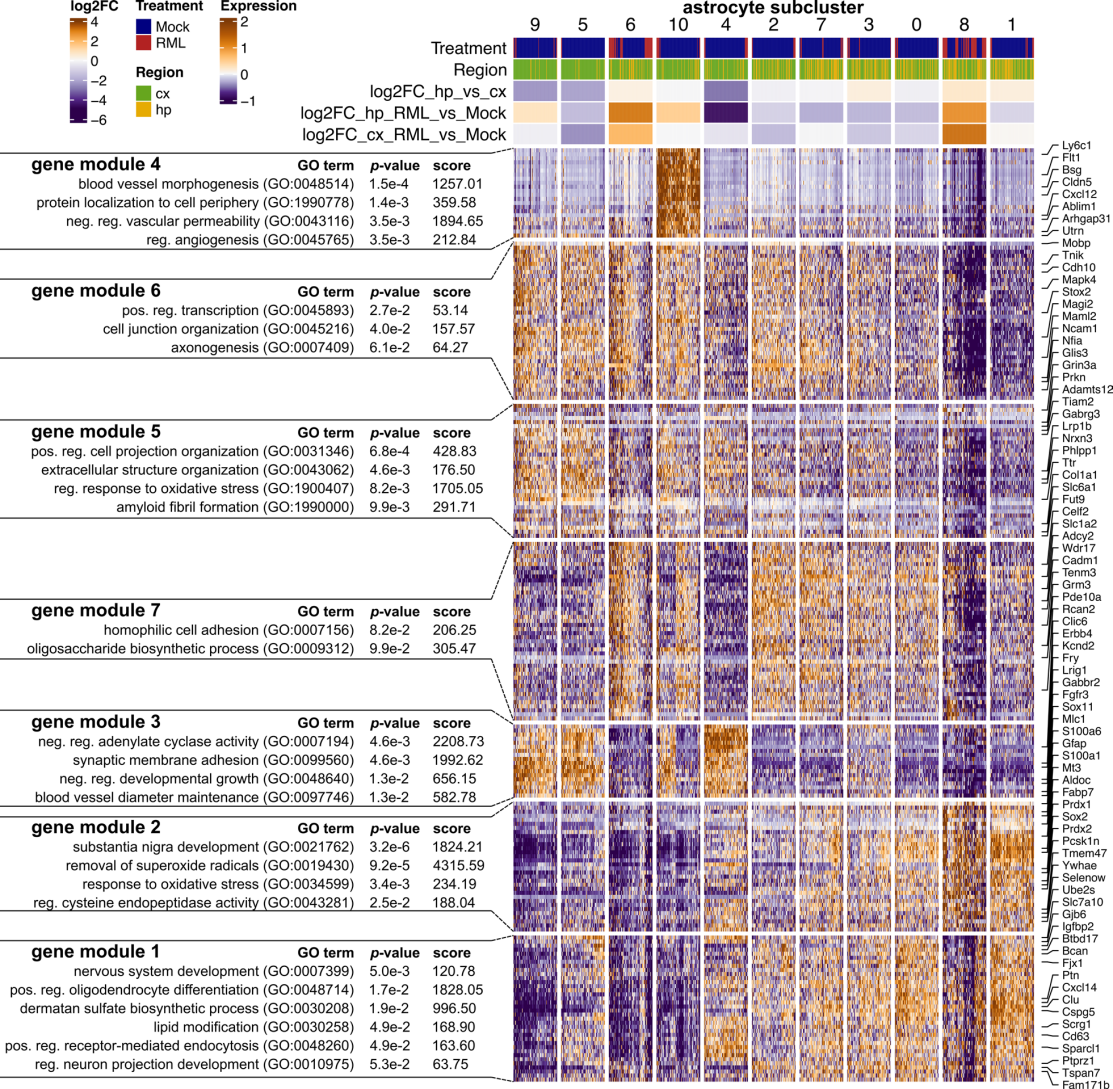
Gene expression profiles of neuroprotective and disease-associated astrocytes. Hierarchical clustered heatmap showing expression of marker genes across different astrocyte sub-clusters. K-means clustering was used to classify marker genes into 7 modules that were functionally annotated by identifying enriched GO terms using Enrichr

To better characterize the population of astrocytes isolated, we performed a sub-cluster analysis by combining all 7,813 astrocyte transcriptomes (from clusters astro.10 and astro.20) and re-clustering into 11 new astrocyte sub-clusters (Fig. 6A). We examined the relative frequency of these astrocyte sub-clusters among all astrocytes and classified them based on whether they were depleted (“disease-depleted”), unchanged, or increased (“disease-associated”) during disease (Fig. 6C, Additional file 6). The majority of the astrocyte sub-clusters decreased in the prion infected brains, but two (astrocyte sub-clusters 6 and 8) increased, and we suspected that these might correspond to a small population of reactive astrocytes. We examined the expression of astrocyte marker genes across the astrocyte sub-clusters (Fig. 6B and Additional file 1: Fig. S3) and noted that disease-associated astrocyte sub-cluster 8 had high expression of *Gfap*, *Aqp4*, *Vim*, and low expression of *Nrxn3*, consistent with reactive astrocytes [19]. To compare disease-depleted with disease-associated astrocyte subpopulations, we next performed hierarchical clustering of all transcripts that were differentially expressed between astrocyte sub-clusters (Fig. 7). K-means clustering was used to classify astrocyte transcripts into 7 gene modules that were differentially abundant across the astrocyte sub-clusters and were enriched in gene ontologies relevant to astrocyte homeostasis functions such as regulating vascular permeability, axonogenesis, synaptic membrane adhesion, removal of superoxide radicals and metabolism (Fig. 7). Disease-depleted astrocyte sub-clusters (0, 2, 3, 4, 5, and 7) varied in expression of these homeostasis-related transcripts indicating that they represent populations of neuroprotective astrocytes. The top transcriptional markers of astrocyte sub-cluster 8 were *S100a6*, *S100a1*, *Prdx1*, and *Hopx* and among these, *S100a6* [7, 29], and *Prdx1* [80] are known to be expressed by disease-associated astrocytes. Astrocyte sub-cluster 8 also had particularly low expression of the homeostatic/neuroprotective gene modules (Fig. 7, gene modules 3, 4, 5, 6, and 7), suggesting loss of neuroprotection by this disease-associated sub-cluster. The top transcriptional markers of astrocyte sub-cluster 6 were *Glis3*, *Cadm1*, *Zbtb20* and *Maml2* (Additional file 1: Fig. S3). *Cadm1* mediates astrocyte-to-astrocyte adhesion [69] while *Zbtb20* promotes astrocytogenesis [56]. However, we did not observe a clear transcriptional profile unique to the disease-associated astrocyte sub-clusters 6 and 8 (Fig. 7), indicating that they were at best, only at the very early stages of becoming reactive. To exclude the possibility of reactive astrocytes clustering together with reactive microglia, we examined canonical astrocyte makers in the sub-clustered microglia dataset and did not observe the presence of transcriptionally distinct reactive astrocytes (Additional file 1: Fig. S4). Populations of astrocytes are well known to be maintained throughout prion disease [74], and we have no reason to suspect decreased astrocyte cell counts within the brain. Therefore, we attribute the decrease in astrocyte relative frequency during prion disease (Fig. 3) to a lack of reactive astrocytes from our dataset. The lack of reactive astrocytes is most likely the result of technical limitations of our approach for preparing single-cell suspensions. For example, reactive astrocytes might have been closely associated with cell debris and removed from the single cell suspensions.

Unfortunately, our live scRNASeq approach did not allow us to assess how different the transcriptomes of strongly activated reactive astrocytes are from those of neuroprotective astrocytes. However, we suspect that the decrease in relative frequency of neuroprotective astrocyte sub-clusters (0, 2, 3, 4, 5, and 7) might indicate that these neuroprotective astrocytes are being converted into a reactive form. The differential expression analysis of astrocyte clusters from the full single cell atlas (Fig. 2B) would suggest that the few astrocytes isolated from the RML infected mice were at the very early stages of becoming reactive with disrupted homeostatic/neuroprotective functions, likely corresponding to astrocyte sub-clusters 6 and 8 identified by the astrocyte sub-cluster analysis (Fig. 7). If this is true, the striking disease-associated depletion of astrocytes from our single-cell atlas (Fig. 3) would imply that nearly all homeostatic astrocytes are converted to a reactive form. However, the technical limitations of our approach prevent us from making this conclusion, and it is possible that single nucleus RNAseq is better suited towards characterizing reactive astrocytes. Therefore, further studies are required to determine how many neuroprotective astrocytes remain non-reactive during disease, and this requires identification of specific markers to delineate between neuroprotective and reactive astrocytes. This would be an interesting line of investigation because observations by others suggest that loss of astrocyte homeostatic functions during prion disease might contribute to neurotoxicity [5, 44]. Indeed, loss of astrocyte homeostasis is a common feature of neurodegeneration [10, 65] and can contribute to neurotoxicity through abnormal EGFR signaling [85], excessive glutamate release/defective glutamate uptake [28, 60, 61, 71], oxidative stress [1], and dysfunctional metabolism [2].

### Transcriptional profiles of neurons during prion disease

Transcriptional changes related to neuronal dysfunction and demise have been challenging to identify using bulk RNAseq data, so we were interested to see how scRNASeq could contribute to defining molecular pathways of cell damage and death in neurons. Firstly we noted that the number of neurons within our dataset was small (8,244/147,536 = 5.6%). This was not surprising as the cellular connectivity and extended processes of neurons may make them particularly vulnerable to cell disruption techniques. However, we identified disease-altered neuronal transcripts involved in synaptic signaling and axon guidance (Fig. 2B, Additional file 8), similar to what is seen in bulk RNAseq [12, 30, 39, 46–49, 72, 73]. There were also altered transcripts of immature neurons that were related to *Cxcl2* production, response to magnesium and exosome biogenesis. As expected, we noticed a trend towards decreased frequencies of mature neurons, while some of the immature neuron populations were increased (Fig. 3).

**Fig. 8:**
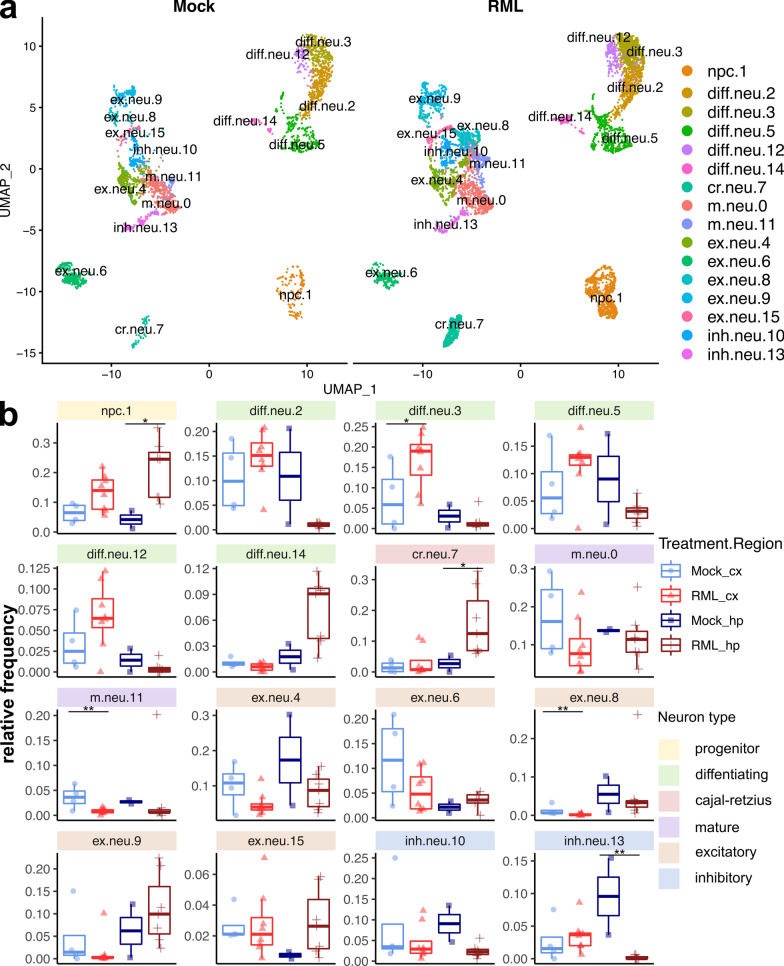
Sub-cluster analysis of neurons reveals immature and mature neuron sub-populations associated with RML disease. **A** UMAP projection of all 8,244 neurons clustered into immature and mature neuron sub-types. **B** The relative proportion of each neuron sub-cluster is plotted for the cortex and hippocampus of RML and mock infected mice. *p* values were calculated using non-parametric Mann–Whitney U tests. * *p* values  < 0.1, ** *p* values  < 0.05, *** *p* values  < 0.01. All *p* values are provided in additional file 7. npc—neural progenitor cell; diff.neu—differentiating neuron; cr.neu—cajal retzius neuron; m.neu—mature neuron; ex.neu—excitatory neuron; inh.neu—inhibitory neuron

To more precisely characterize neuronal subpopulations that differentially respond to prion disease, we collapsed all mature and immature neuron transcriptomes (g.neu.16, g.neu.31, g.neu.33, g.neu.34, im.neu.18, im.neu.24 and im.neu.32) and re-clustered the 8,244 cells into 16 transcriptionally distinct clusters (Fig. 8A). We categorized these clusters based on the abundance of marker genes for neural progenitor cells (*Mki67*), immature differentiating neurons (*Dcx*), cajal-retzius neurons (*Reln*), mature neurons (*Rbfox3*), excitatory neurons (*Slc17a7*) and inhibitory neurons (*Gad1*) (Fig. 8A and Additional file 1: Fig. S5). Clusters m.neu.0 and m.neu.11 were categorized as mature neurons because they did not express clear markers of either excitatory or inhibitory neurons. We also examined the expression of *Prnp* and found it to be most highly expressed by excitatory neurons (Additional file 1: Fig. S5).

**Fig. 9:**
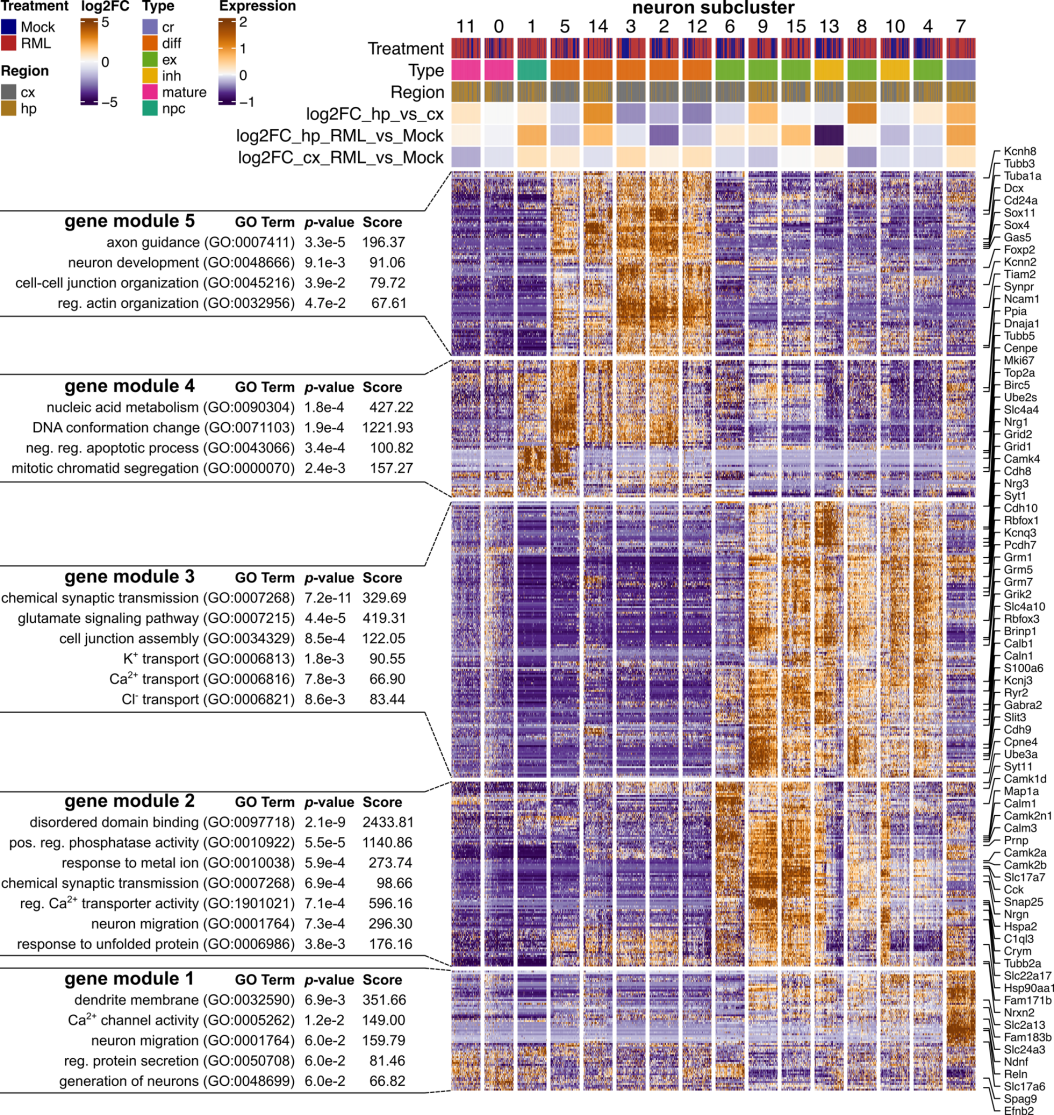
Gene expression profiles of mature and immature neuron sub-clusters associated with RML disease. Hierarchical clustered heatmap showing expression of marker genes across neuronal sub-clusters. K-means clustering was used to classify marker genes into 5 modules that were functionally annotated by identifying enriched GO terms using Enrichr. cx—cortex; hp—hippocampus; cr—cajal retzius neuron; diff—differentiating neuron; ex—excitatory neuron; inh—inhibitory neuron; npc—neural progenitor cell

We next compared the relative frequency of the cell populations within the sub-clustered neuronal dataset and observed several cellular composition differences associated with prion disease (Fig. 8B, Additional file 7). Furthermore, we compared neuronal gene expression profiles via hierarchical clustering of all identified marker genes for each neuronal sub-cluster (Fig. 9). K-means clustering was used to classify these transcriptional markers into 5 gene modules and each neuron subtype expressed gene modules enriched with relevant functional ontologies. Neural progenitor cells expressed cell cycle genes, differentiating neurons expressed axon guidance genes and mature neurons primarily expressed synaptic signaling genes (Fig. 9).

### Abnormal neurogenesis during prion disease

The relative frequency of neural progenitor cells increased in association with RML disease, especially in the hippocampus (Fig. 8B). This was consistent with a number of studies that have detected increased neurogenesis in the hippocampus [20, 22] which may protect against prion disease [34]. Relatively few transcripts of neural progenitors were differentially expressed (Fig. 2, cluster im.neu.24), although we noted the top overexpressed transcripts were *Spp1* and *Lpl*, and the top underexpressed transcripts was *Csmd3*. We also identified 5 clusters of immature differentiating neurons, some of which appeared to increase or decrease in association with prion disease (Fig. 8B), although only cluster diff.neu.3 achieved statistical significance (increased in the cortex). Therefore, this analysis was unable to resolve whether any of the differentiating neuron sub-clusters were associated with disease. We did however notice a number of differentially expressed transcripts of differentiating neurons, both within the full single cell atlas (Fig. 2, cluster im.neu.18) and within individual sub-clusters from the sub-cluster analysis (Additional file 1: Fig. S6). Upregulated transcripts of differentiating neurons in the full single cell atlas (cluster im.neu.18) were related to axonogenesis (*Hsp90aa1*, *Dab1*, *Auts2*, *Dcc*, *Nrxn1*, *Ntn4*, *Ncam1*, *Ank3*, *Cntn4*), potassium transport (*Slc24a2*, *Kcnt2*, *Kcnd3*, *Kcnh7*, *Kcnb2*, *Kcnq5*, *Kcnn2*) and synapse organization (*Cacnb4*, *Cdh2*, *Nrxn1*, *Ank3*, *Snca*), together implicating possible modulation of neuronal differentiation. Other studies have shown that PrP^Sc^ can replicate in *Dcx*^+^ immature neurons, resulting in impaired differentiation [68] and that newborn neurons differentiate abnormally in prion infected mice [22]. Thus, the disease-associated transcription of differentiating neurons might represent dysfunction, possibly explaining why neurogenesis is ultimately unsuccessful at mitigating neuronal loss during prion disease.

We were also surprised to observe increased relative frequencies of cajal-retzius neurons in the hippocampus of RML infected mice (Fig. 8B). One of the primary functions of cajal-retzius neurons is secretion of Reelin (*Reln*), a protein that regulates neuron migration during neurogenesis [13]. It is therefore possible that cajal-retzius neurons are involved in abnormal hippocampal neurogenesis during prion disease. Very few transcripts of cajal-retzius neurons were differentially expressed (Fig. 2, cluster im.neu.32), so we have no reason to suspect that these cells were dysfunctional. We are not aware of any studies that have examined cajal-retzius neurons during prion disease, but they are reportedly decreased in the hippocampus due to apoptosis in an Alzheimer’s disease mouse model [88]. This might indicate that increased numbers of cajal-retzius cells are distinguishing feature of prion disease. Of course, here we only present relative frequencies, and it is possible that the increase was due to technical reasons, such as resistance of cajal-retzius neurons to death during our cell isolation protocol.

### Differential response of excitatory and inhibitory neurons to prion disease

In the sub-clustered neuronal dataset, many excitatory neurons were generally enriched in either the hippocampus or cortex, and were not strongly affected at the population level by RML disease (Fig. 8B). Clusters ex.neu.9 and ex.neu.15 even trended towards a slight increase in the hippocampus of RML infected mice. We also identified populations of inhibitory neurons that were generally decreased in the hippocampus of RML infected mice (Fig. 8B). The trends towards slightly increased excitatory neurons and decreased inhibitory neurons in the hippocampus is consistent with previous histopathological analyses showing inhibitory neurons to be more vulnerable to prion infection [8, 24–26]. We found that excitatory neuron populations sustained during disease (ex.neu.9 and ex.neu.15) had high expression of gene module 2, which was expressed at much lower levels in the inhibitory neuron populations that appeared more sensitive to RML disease (inh.neu.10 and inh.neu.13) (Fig. 9). Gene module 2 was enriched in ontologies related to neuron maintenance, such as disordered domain binding, regulation of phosphatase activity, response to metal ion and response to unfolded protein. We were also surprised to see that *Prnp* was among gene module 2, and was more highly expressed by the neurons that appeared more resistant to prion infection compared to those that appeared more sensitive.

Sub-cluster analysis of the excitatory and inhibitory neurons did not reveal any distinct clusters of transcriptomes that were associated with disease, and so we instead examined disease-associated transcription through differential expression analysis. Within the full single cell atlas, the main cluster of mature neurons (g.neu.16) had the largest number of differentially expressed transcripts out of any cell cluster with 168 upregulated and 128 downregulated. These disease-altered transcripts seem to hint at synaptic dysfunction. Notable upregulated transcripts of cell cluster g.neu.16 were related to axonogenesis (*Robo2, Hsp90aa1, Epha6, Dcc, Sema6d, Nrxn3, Unc5d, Ank3, Kif5c, Tubb3, Map1b, Fez1, Ncam1, Slit2, Dscaml1*), regulation of cation transport (*Camk2b, Cacnb4, Dlg2, Lrrc7, Camk2a, Kcnab1, Ank2, Ank3, Grin2b, Shisa6*), and synaptic transmission (*Gabra2, Dtna, Syt1, Grid1, Grik2, Grin2b, Shisa6, Nrgn, Cacnb4, Dlg2, Grm7, Npy, Slc17a6, Asic2, Erc2, Dlgap2*). Similar types of neuronal transcripts were also downregulated in cluster g.neu.16, including those related to synaptic transmission (*Gabrb3, Snap25, Gabrb2, Gabbr2, Grid2, Ptprn2, Kcnd2, Nrxn1, Lin7a, Grik1, Cdh8, Rims1, Dlgap1, Gria3, Gria4*), neuronal projections (*Epha4, Ntrk2, Cdh2, Fut9, Il1rapl1, Nptn, Ndnf, Ctnna2, Tox, Pak3*) and axonogenesis (*Epha4, Dab1, Lama1, Dok6, Nrxn1, Nptn, Nrcam, Cck, Ctnna2, Efna5, Pak3*). Within the full single cell atlas, excitatory and inhibitory neuronal subtypes were not completely resolved. Therefore, we also performed a differential expression analysis between cells isolated RML and Mock infected mice within each cluster in the sub-clustered neuronal dataset (Additional file 1: Fig. S6). Many of the differentially expressed transcripts originated from excitatory neuron populations and were related to synaptic transmission, although there were also differentially expressed transcripts in inhibitory neuron and differentiating neuron populations. This raises the possibility of synaptic dysfunction within the excitatory neurons that were not depleted in our dataset. A few of these synaptic transcripts were also altered in inhibitory neurons, but we noted a group of disease altered transcripts of inhibitory neurons that was not altered in excitatory neurons. These transcripts were enriched in ontologies related to potassium transport (*Ank2, Atp1b2, Atp1b1*) and calcium homeostasis (*Calm3, Tmtc2, Ank2, Atp1b1, Snca*), implying a distinct response of inhibitory neurons. Given that we were unable to resolve genuine disease-associated clusters of neuronal transcriptomes, a larger single-cell dataset of neurons is warranted to further investigate selective vulnerability and to better define molecular disruptions to neurons during disease.

## Discussion

We report an extensive library of 147,536 single cell transcriptomes from matched tissue samples of prion infected and non-infected mice. Whilst previous studies rely on bulk RNAseq to measure average transcript abundances within a tissue [12, 30, 39, 46–49, 70, 72, 73], here we profiled transcript-level and cell-population level responses to prion disease. To minimize cell-composition complexity and to target brain regions particularly affected during prion disease, we chose the murine cortex and hippocampus for this study. The data provides further resolution of the pathobiology of disease and some of the more striking findings were the apparent dysfunction of homeostatic astrocytes and vascular cells, the diversity of reactive microglia and differential response of neuronal populations. This represents a new technological advance with huge potential for uncovering the molecular basis for pathological changes within the prion-infected brain at the cellular subtype level.

Our single cell differential gene expression correlates in many respects with previous bulk RNAseq datasets of prion infection [12, 30, 39, 46–49, 70, 72, 73]. Glia mount the most prominent phenotypic response to infection, and this was readily observable in our dataset. Our analysis precisely tracked transcriptional changes within individual subpopulations of brain cells and distinguished brain cell subtypes that were associated with prion disease. Nonetheless some limitations exist with this approach including biased selection for certain brain cell types during preparation of single cell suspensions, as well as any transcriptional changes that might occur during the process of tissue dissociation. Thus, the cell populations profiled here likely do not fully reflect brain cells in their natural state during prion disease. Other approaches to analyze individual cell types, such as single nuclei sequencing, and ribosomal profiling have their own associated technical challenges [4, 9, 21, 86], and we believe multiple approaches are necessary to fully describe molecular and cellular changes in the prion infected brain. Given that we have identified a number of unique cell type clusters, the next steps will be to perform validation of these cell types within the brain and determine the interplay between the different sub-populations and replicating prions.

We characterized 11 sub-clusters of transcriptionally distinct microglia that differentially express functional markers of homeostasis, inflammatory cytokine signaling, phagocytosis, and antigen presentation. Based on expression of these functional markers, and consistent with observations from single cell RNAseq studies of Alzheimer’s disease [16], we described 5 subtypes of microglia including: (1) homeostatic, (2) proliferating, (3) phagocytic, (4) type I interferon responding (IFN) and (5) antigen presenting (MHC). Phagocytic, proliferating, IFN and MHC microglia subtypes corresponded to reactive microglia and were associated with disease through increased relative frequency within the RML infected mice. The microglia from our study were similar to those seen in a recent single-cell RNAseq study of human Alzheimer’s disease patients [59], raising the possibility that they are relevant to disease in humans. Specific genes such as *Il12b*, *Serpine1*, *Jun*, *Ftl1*, *Nav2*, *Cd14*, *Trim30a* and *Cd74* demarked some of the microglial subtypes—possibly serving as markers of functionally diverse microglial subsets. Therefore, the next steps will be to verify these microglial sub-populations throughout disease progression and to determine their relative contribution to the reported protective and detrimental properties of activated microglia during prion disease [53, 58, 63].

In contrast to previous bulk RNAseq analyses of prion infected brain tissue that primarily identify inflammatory gene expression [12, 30, 39, 46–49, 72, 73], disruptions to brain homeostasis were much more apparent in our single cell dataset. This includes the dysregulation of homeostatic/neuroprotective astrocyte gene expression, decrease in relative frequency of homeostatic microglia, transcriptional dysfunction of vascular cell populations that make up the blood brain barrier, modulation of oligodendrocyte progenitor cells and abnormal neurogenesis. These details provide additional context towards understanding neuronal dysfunction and demise in prion disease, given that loss of astrocyte neuroprotection in particular can result in neurotoxicity [1, 2, 28, 60, 61, 71, 85]. Moreover, single cell studies of Alzheimer’s disease have found disruptions to homeostatic astrocytes and vascular cells to be important components of pathogenesis [45, 51]. From this, it is apparent that restoring brain homeostasis will be an important consideration for developing therapeutics against prion disease, in addition to removal of the disease-causing agent and attenuation of excess inflammatory signaling.

Interestingly, we observed increased relative frequency of proliferating cell populations in the prion-infected brain, including neural progenitor cells, oligodendrocyte progenitor cells and proliferating microglial subtypes. While the relative frequencies presented here do not equate to true measurements of absolute cell count, previous studies have implicated proliferation of microglia [74] and neural progenitor cells [20, 22] during prion disease. We speculate that a common means exists to promote cell proliferation during prion disease—possibly in an unsuccessful attempt to restore brain cells that are lost during disease. Indeed, activated microglia are known secrete factors that promote oligodendrocyte progenitor cell proliferation [83] and enhance neurogenesis [18, 57, 64]. Alternatively, PrP^C^ can modulate both proliferation of oligodendrocyte progenitor cells [11] and neurogenesis [62, 77]—raising the possibility that lack of functional PrP^C^ owing to prion replication could contribute to increased cell proliferation. Therefore, uncovering exactly how neural progenitor cells are modulated during prion disease would be an interesting approach that could help inform strategies to restore dying neurons and repair damage in the diseased brain.

In conclusion, single-cell RNAseq represents a comprehensive approach to characterize transcript-level and cell-composition changes throughout prion disease. We identified numerous disease-associated cellular subpopulations that warrant further validation, particularly in the case of microglia. Our analysis highlights the complexity of the glial and neuronal reactome to prion replication and accumulation. Future applications of the data will be to identify specific transcriptional markers that distinguish pathological cell phenotypes and to gain further molecular insight into the disruptions that underlie neurodegenerative progression in prion diseases. This includes discriminating sup-populations of disease-responding cells as either neuroprotective or driving pathology. Also important will be defining commonalities and differences between disease processes of various degenerative diseases. Overall, this dataset, and others like it, provide higher resolution in the journey to unravel the complex dysregulation occurring in different brain cell types throughout neurological disease during prion infection.

## Supplementary Information

**Additional file 1**: Supplementary tables and figures.

**Additional file 2**: Globally distinguishing transcriptional markers of each cell cluster within the full single-cell atlas. Only positive markers were reported, identified via criteria of |log2FC| > 0.5, FDR *p*-value < 0.05 and %cell expression difference > 0.1.

**Additional file 3**: Total number of cells isolated from RML or Mock infected mice that were classified into each of the cell clusters from the full single cell RNAseq atlas.

**Additional file 4**: Cell type identity scores calculated using SCType for each cell cluster in the full single cell RNAseq atlas.

**Additional file 5**: Mann-Whitney *p*-values corresponding to relative frequency differences between RML and mock infected mice for each cell cluster in the full single cell RNAseq atlas.

**Additional file 6**: Mann-Whitney *p*-values corresponding to relative frequency differences between RML and mock infected mice for each cell sub-cluster in the subset astrocyte dataset.

**Additional file 7**: Mann-Whitney *p*-values corresponding to relative frequency differences between RML and mock infected mice for each cell sub-cluster in the subset neuron dataset.

**Additional file 8**: Differentially expressed transcripts between cells isolated from RML and Mock infected mice within each cell cluster in the full single cell RNAseq atlas. Differentially expressed transcripts were defined by criteria of FDR *p*-value < 0.05, |log2FC| > 0.5 and %cell-expression difference > -0.1 or < 0.1 for increased/decreased genes respectively.

**Additional file 9**: Mann-Whitney *p*-values corresponding to relative frequency differences between RML and mock infected mice for each cell sub-cluster in the subset microglia dataset.

## Data Availability

The single-cell RNAseq dataset is available from the Broad Institutes’ Single Cell Portal at: https://singlecell.broadinstitute.org/single_cell/study/SCP1962/dysregulation-of-neuroprotective-astrocytes-a-spectrum-of-microglial-activation-states-and-altered-hippocampal-neurogenesis-are-revealed-by-single-cell-rna-sequencing-in-prion-disease.

## Code availability

All R code used to perform the analysis is available from the GitHub repository at: https://github.com/jslota/scRNAseq-prion-infection.
