# Supplementary material for: Dysregulation of neuroprotective astrocytes, a spectrum of microglial activation states, and altered hippocampal neurogenesis are revealed by single-cell RNA sequencing in prion disease
Source: Acta Neuropathol Commun. 2022 Nov 9;10:161. doi: 10.1186/s40478-022-01450-4 (PMC9647949; doi:10.1186/s40478-022-01450-4)
Supplement: Supplementary file 1 — Additional file 1: Supplementary tables and figures. [file 40478_2022_1450_MOESM1_ESM.docx]

**Supplementary Table 1 – Brain region, treatment, and dpi of all single cell sequencing libraries used in the study.**

| **Mouse No.** | **Region** | **Treatment** | **dpi** |
| --- | --- | --- | --- |
| mouse25 | hp | Mock | 110 |
| mouse48 | cx | Mock | 147 |
| mouse48 | hp | Mock | 147 |
| mouse60 | cx | Mock | 168 |
| mouse61 | cx | Mock | 186 |
| mouse73 | cx | Mock | 189 |
| mouse122 | cx | RML | 152 |
| mouse122 | hp | RML | 152 |
| mouse132 | cx | RML | 162 |
| mouse132 | hp | RML | 162 |
| mouse133 | cx | RML | 161 |
| mouse133 | hp | RML | 161 |
| mouse134 | cx | RML | 165 |
| mouse138 | cx | RML | 166 |
| mouse138 | hp | RML | 166 |
| mouse140 | cx | RML | 172 |
| mouse140 | hp | RML | 172 |
| mouse142 | cx | RML | 159 |
| mouse142 | hp | RML | 159 |
| mouse145 | cx | RML | 169 |
| mouse145 | hp | RML | 169 |

*hp – hippocampus; cx - cortex*


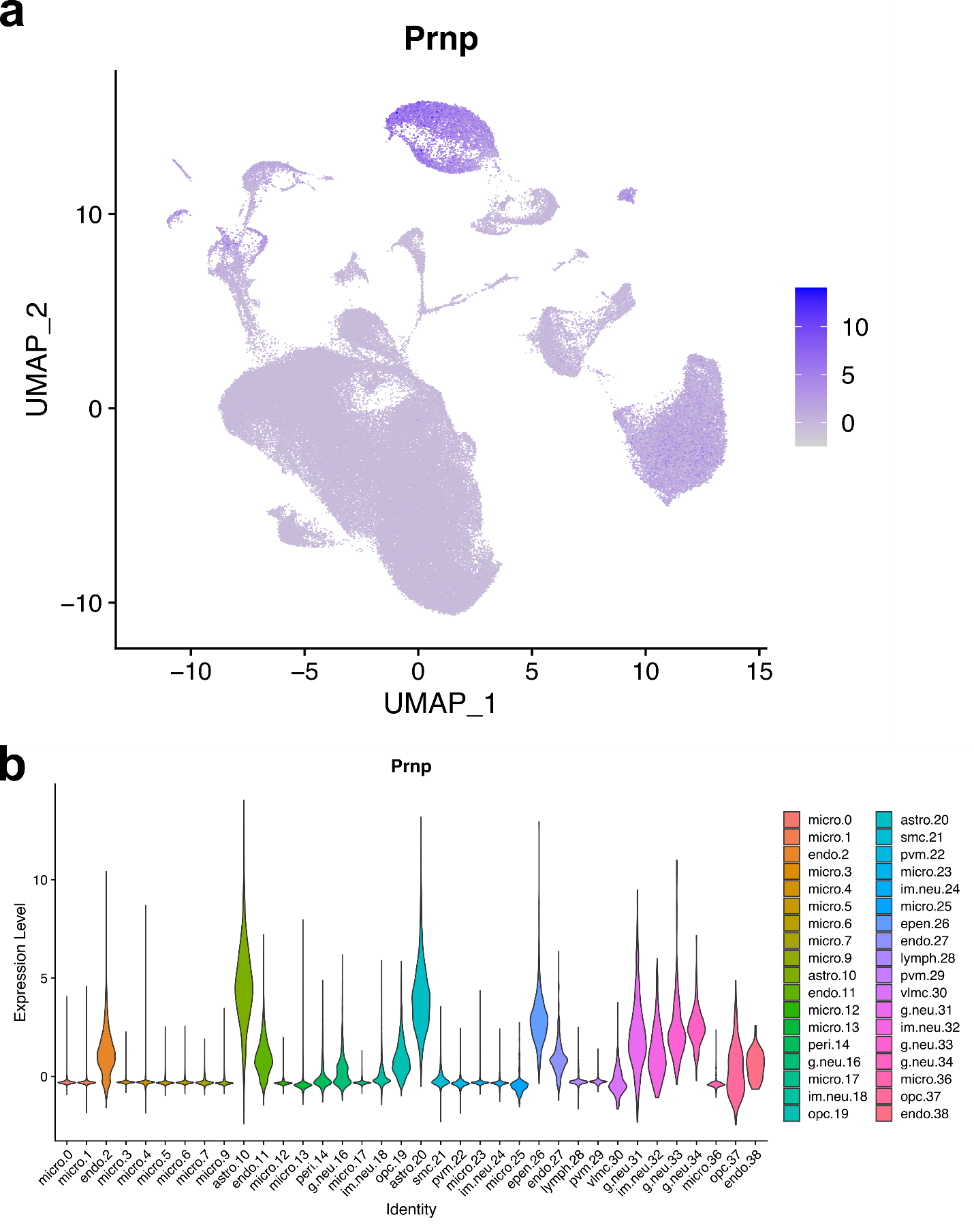


**Supplementary Figure 1. Expression of *Prnp* across different brain cell subsets. (A)** UMAP projection plot with color mapped to *Prnp* expression. **(B)** Violin plot showing *Prnp* expression across brain cell sub-clusters.

**
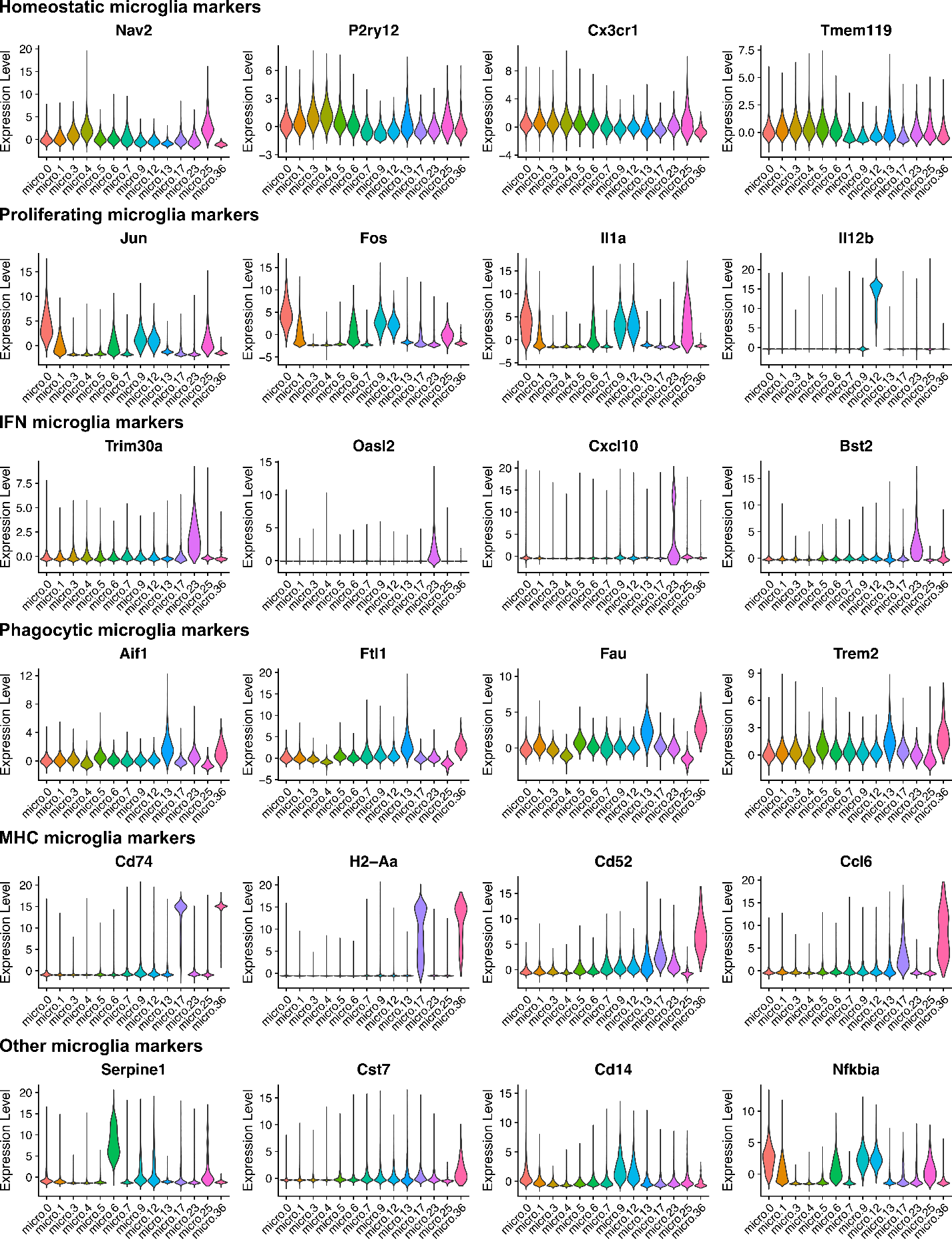
**

**Supplementary Figure 2. Expression of marker genes in microglial sub-clusters.** Violin plots show expression across microglial sub-clusters for top transcriptional markers of homeostatic, proliferating, type I interferon responsive (IFN), phagocytic, and antigen presenting (MHC) microglia.

**
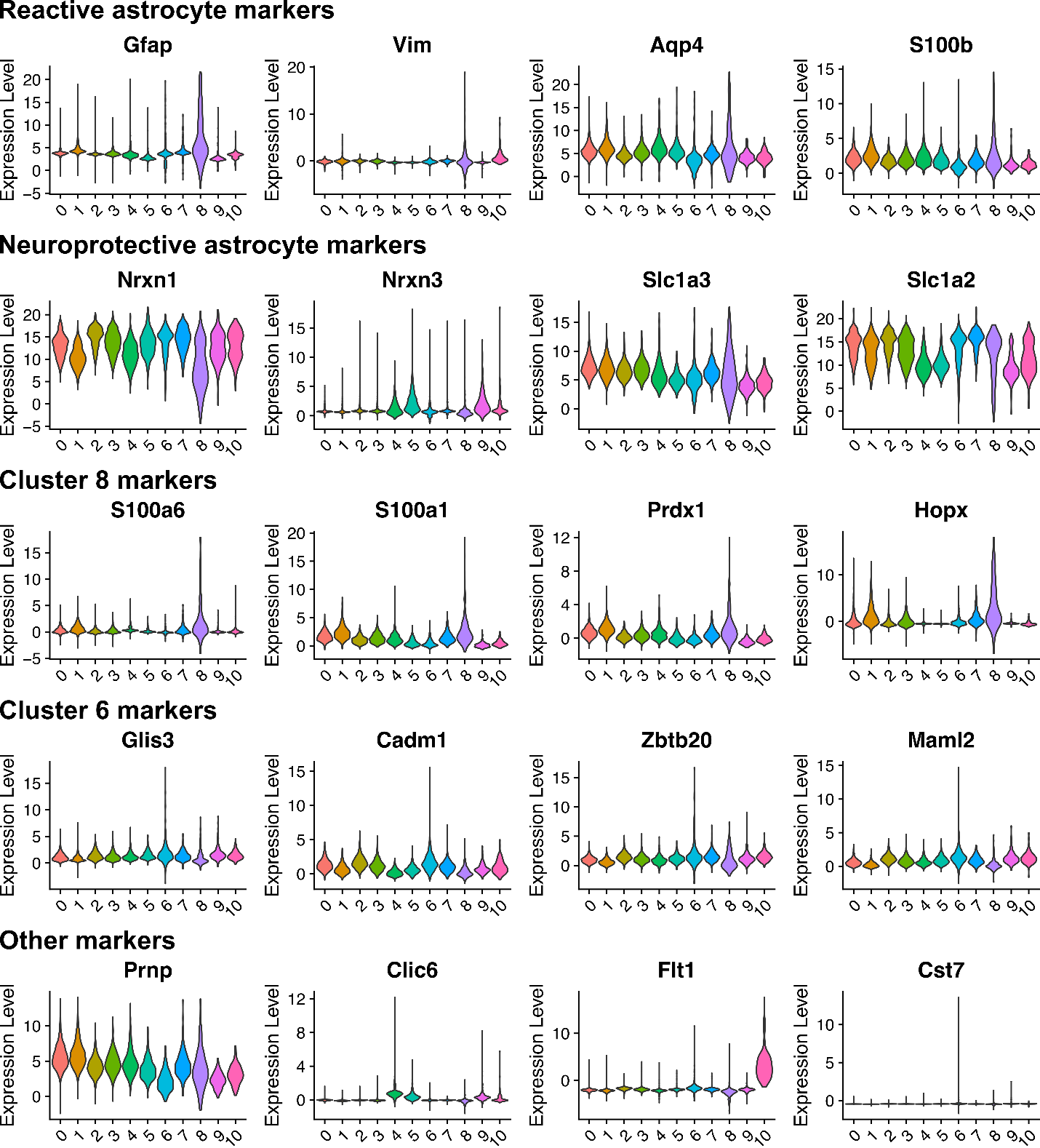
**

**Supplementary Figure 3. Expression of marker genes in astrocyte sub-clusters.** Violin plots show expression across astrocyte sub-clusters for transcriptional markers of reactive and neuroprotective astrocytes, as well as top markers for disease-associated astrocyte sub-clusters 6 and 8.

**
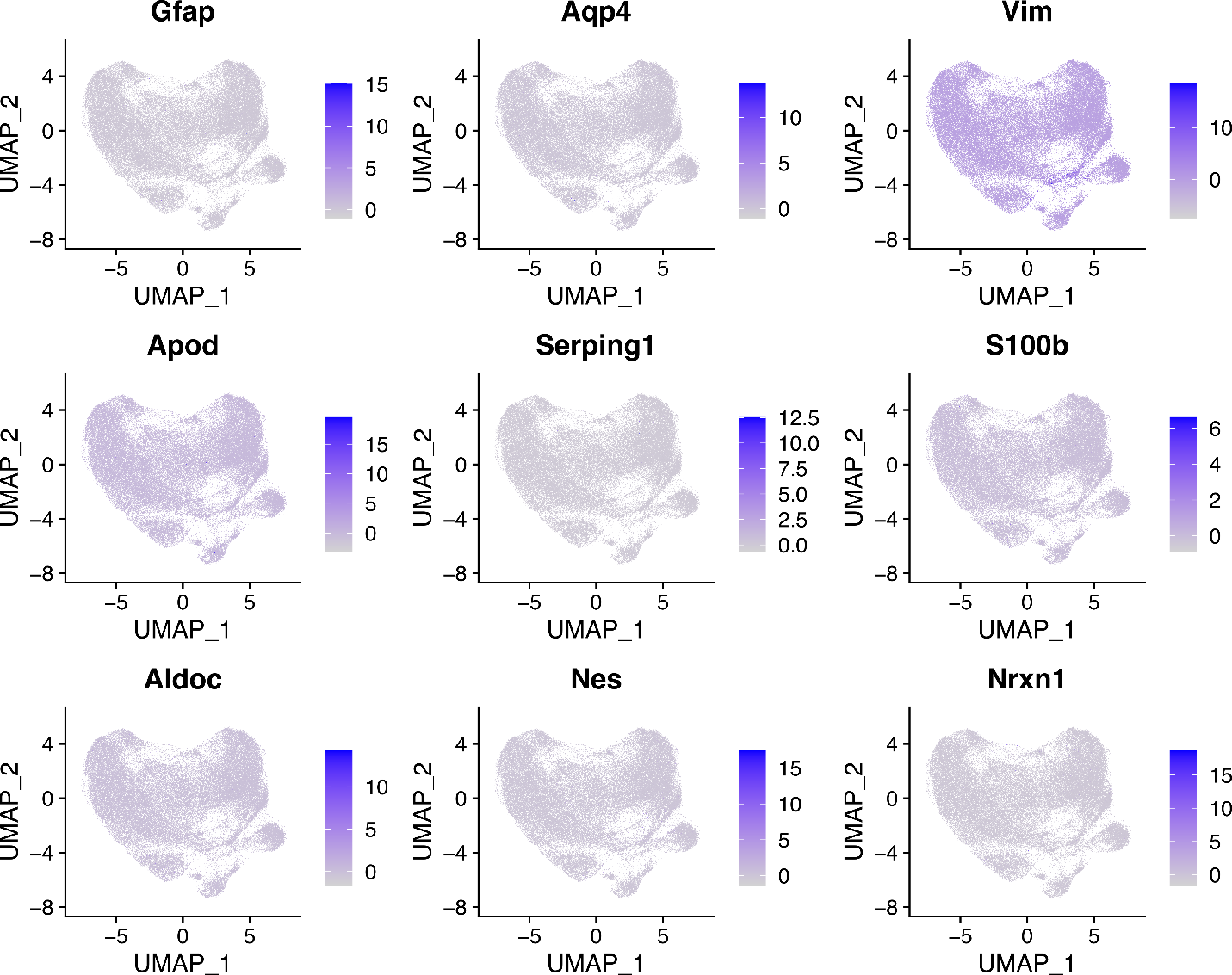
**

**Supplementary Figure 4. Expression of astrocyte markers in sub-clustered microglia dataset.** UMAP projection plots of the sub-clustered microglia dataset with color mapped to expression of select reactive astrocyte transcriptional markers.


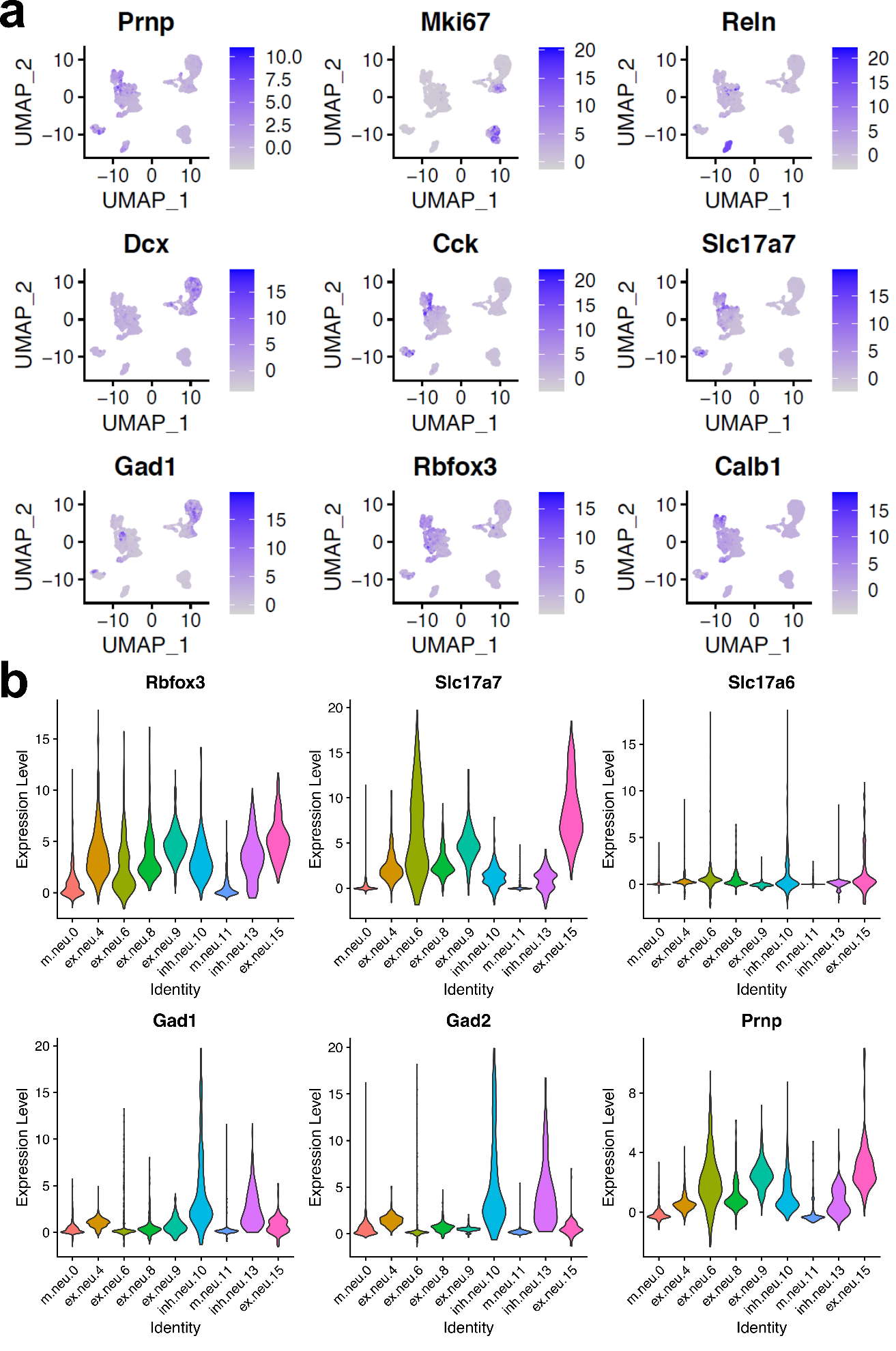


**Supplementary Figure 5. Expression of marker genes across neuron sub-clusters. (A)** Expression of mature and immature neuronal marker genes on UMAP projection plots. **(B)** Violin plots showing expression of excitatory and inhibitory neuronal markers across mature neuron clusters.


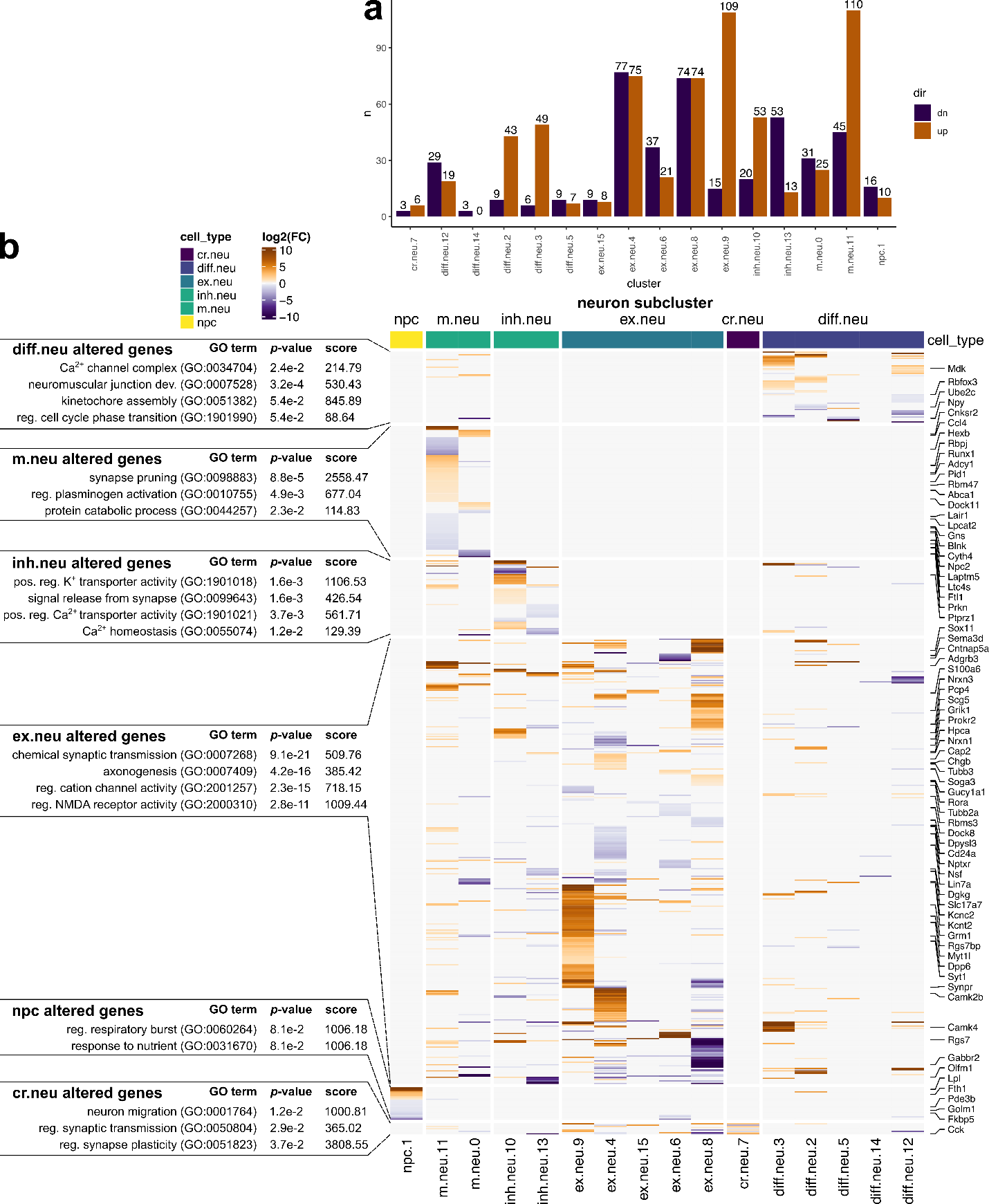


**Supplementary Figure 6. Differentially expressed transcripts within each neuron sub-cluster between cells isolated from RML and Mock infected mice. (A)** Number of transcripts that met differential expression criteria within each cluster when comparing cells isolated from RML and Mock infected mice. Differentially expressed transcripts were defined by: FDR *p*-value < 0.05, |Log2 fold change| > 0.5, > 25% cell expression and 10% increased/decreased cell expression for increased/decreased transcripts respectively. **(B)** Hierarchical clustering of Log2 fold changes for all differentially expressed transcripts within each cluster that were associated with RML infection in a previous study employing the same mouse model. *npc – neural progenitor cell; diff.neu – differentiating neuron; cr.neu – cajal retzius neuron; m.neu – mature neuron; ex.neu – excitatory neuron; inh.neu – inhibitory neuron.*
